# The Aurora kinase B relocation blocker LXY18 triggers mitotic catastrophe selectively in malignant cells

**DOI:** 10.1371/journal.pone.0293283

**Published:** 2023-10-30

**Authors:** Julia Kalashova, Chenglu Yang, Hongmei Li, Yan Long, Duo Yu, Ting Zhang, Xumei Liu, Namrta Choudhry, Qiong Shi, Thaddeus D. Allen

**Affiliations:** 1 Division of Discovery Oncology, Chengdu Anticancer Bioscience, Chengdu, Sichuan, China; 2 Department of Basic Cancer Research, J. Michael Bishop Institute of Cancer Research, Chengdu, Sichuan, China; Lahore University of Management Sciences, Syed Babar Ali School of Science and Engineering, PAKISTAN

## Abstract

The mitotic regulator, Aurora kinase B (AURKB), is frequently overexpressed in malignancy and is a target for therapeutic intervention. The compound, LXY18, is a potent, orally available small molecule that inhibits the proper localization of AURKB during late mitosis, without affecting its kinase activity. In this study, we demonstrate that LXY18 elicits apoptosis in cancer cells derived from various indications, but not in non-transformed cell lines. The apoptosis is p53-independent, triggered by a prolonged mitotic arrest and occurs predominantly in mitosis. Some additional cells succumb post-mitotic slippage. We also demonstrate that cancer cell lines refractory to AURKB kinase inhibitors are sensitive to LXY18. The mitotic proteins MKLP2, NEK6, NEK7 and NEK9 are known regulators of AURKB localization during the onset of anaphase. LXY18 fails to inhibit the catalytic activity of these AURKB localization factors. Overall, our findings suggest a novel activity for LXY18 that produces a prolonged mitotic arrest and lethality in cancer cells, leaving non-transformed cells healthy. This new activity suggests that the compound may be a promising drug candidate for cancer treatment and that it can also be used as a tool compound to further dissect the regulatory network controlling AURKB localization.

## Introduction

Aurora kinase B (AURKB) orchestrates multiple key steps of cell division, including initiating mitotic entry, facilitating chromosome movement, maintaining activation of the spindle assembly checkpoint (SAC), organizing the central spindle and midbody, and facilitating the completion of cytokinesis [1, 2]. These diverse functions of AURKB are mediated by its kinase activity and executed as part of a larger protein complex, the chromosomal passenger protein (CPP) complex [3]. The CPP complex also includes three other subunits, inner centromere protein (INCENP), Survivin, and Borealin [4]. To fulfill its versatile mitotic functions, the CPP complex relocates dynamically in a highly coordinated manner as mitosis proceeds. For example, relocation of the CPP from chromosomes to the spindle midzone, and therefore repositioning AURKB catalytic activity, occurs at the onset of anaphase. AURKB activity eventually concentrates at the midbody in telophase [5].

AURKB is often overexpressed in human malignancy and elevated AURKB is associated with high histologic grade and unfavorable outcome in different types of cancers [6]. In addition, ectopic overexpression of AURKB can transform cells *in vitro* and elicit tumorigenesis in animal models [7]. AURKB has, therefore, been investigated extensively as a drug target for cancer therapy. Several AURKB inhibitors have progressed into clinical trials [8, 9]. Unfortunately, the majority of compounds did not progress to late-stage trials due to dose-limiting toxicity and low response rate [8, 9].

The small molecule AURKB inhibitors used in early trials were type I or II kinase inhibitors, which actively engage the catalytic site [10]. This is a highly conserved region, even among different kinase families and, not surprisingly, most AURKB inhibitors exhibit pan-AURK inhibitory activity. This may have contributed to failure to progress to late-stage trials. Inhibition of AURKA, for example, has been reported to cause unwanted immune suppression [11]. Cross-inhibition of tyrosine kinases outside the AURK family is also typical for AURKB inhibitory compounds and is found causative in undesirable side effects [12]. Finally, AURKB catalytic inhibition itself can have undesired effects, as these compounds can disable AURKB-regulated processes in non-dividing cells [13]. To minimize unwanted effects, new approaches to targeting AURKB or other components of the CPP pathway are needed. This would represent a new therapeutic approach to targeting dividing cells that could address unmet needs in cancer treatment.

One class of compound that could potentially address this need, is 4-phenoxy-quinoline-based compounds which disrupt the mitotic localization of AURKB [14]. This group of bioactive compounds was identified from a high throughput, mechanism-informed phenotypic screening (MIPS) assay. The purpose of the screen was to identify compounds that mimic loss of AURKB function without inhibiting its kinase activity. The most potent compound in this class is N-(3-((6-bromoquinolin-4-yl)oxy)-5-methoxyphenyl)acetamide, also known as LXY18 [15]. This compound blocks the relocation of AURKB during the onset of anaphase with nanomolar potency. The compound also possesses desirable oral pharmacokinetic (PK) properties [16] and tumor-suppressive activity in experiments involving murine xenografts [15]. LXY18 elevates the mitotic index, induces the accumulation of multinucleated cells, and elicits apoptosis *in vitro* and *in vivo*. Elevation of the mitotic index by LXY18 is intriguing because inhibition of AURKB is known to alleviate mitotic arrest by compromising the maintenance of the spindle assembly checkpoint (SAC) response [17]. LXY18, therefore demonstrates a unique mode of action. Instead of enabling mitotic slippage, LXY18 elicits a sustained mitotic arrest to attack cancer cells. This mechanism of action is not consistent with AURKB inhibition alone and consistently, two cancer cell lines, Calu-6 and Hela, refractory to AURKB kinase inhibitors, were sensitive to LXY18.

Here, we demonstrate that LXY18 selectively killed transformed cells as opposed to non-transformed cells. It did so despite our observation of an LXY18-induced inhibition of cytokinesis in both malignant and non-malignant cells. With treatment of malignant cells, nearly all cells were eliminated through apoptosis which emerged after a prolonged mitotic arrest. Only a few escaped from mitotic death. However, these cells also perished shortly after their mitotic slippage or after cytokinetic failure. Both mitotic arrest and apoptosis coincided with an AURKB relocation failure. Mislocalization could not be attributed to blockade of the catalytic activities of MKLP2, NEK6, NEK7 or NEK9, the known regulators of AURKB relocation at the metaphase to anaphase transition. Non-transformed cells were not susceptible to apoptosis and when there was a cytokinetic failure, they underwent a proliferative arrest.

Overall, we found that LXY18 was more potent than AURKB kinase inhibitors in a panel of human cancer cell lines, despite appearing to work, at least in part, through CPP complex mislocalization. This study characterizes LXY18 as a novel anticancer agent that blocks cell division and triggers cell death selectively in malignant cells. LXY18 possesses properties that differentiate it from small molecule mitotic kinase inhibitors and may make it a viable treatment for human malignancy.

## Material and methods

### Cell culture

A human retinal pigment epithelial cell line, RPE A19, and all human cancer cell lines used in this study were purchased from the American Type Culture Collection (ATCC). Following the vendor’s instruction, all cell lines were cultured in either DMEM (Gibco, Cat. No. 12100061) or RPMI-1640 (Gibco, Cat. No. C11875500BT) supplemented with 10% fetal bovine serum (Excell, Cat. No. FSP500 10099141), penicillin (100 U/mL)-streptomycin (100 μg/mL) (Gibco, Cat. No.15140-122), 2 mM L-glutamine (Gibco, Cat. No. 25030081), and 1 mM sodium pyruvate (Gibco, Cat. No. 11360070). Cell culture was maintained in a humidified incubator with 5% CO2 at 37°C. Mycoplasma contamination of each cell line was evaluated by using a PCR-based Myco-LumiTM Luminescent Mycoplasma Detection Kit (Beyotime, Cat. No. C0298M) before experiments were initiated and at the end of the project. Each cell line was amplified and stored in aliquots when it was acquired. Each aliquot was used only for 10–15 passages in order to minimize the risk of genetic drift and ensure consistency in the experimental data.

### Trypan blue exclusion assay of cell viability

The Trypan blue exclusion assay uses Trypan blue dye to selectively stain dead but not living cells. Cells were harvested by trypsinization and suspended in sterile 0.01M PBS (phosphate-buffered saline). A solution of 0.4% Trypan blue dye was mixed with the cell suspension in a 1:1 ratio (v/v) before the sample was loaded into a Hemocytometer chamber (XB-K-25, SMIC). Under an inverted tissue culture microscopy, Trypan blue-positive and negative cells were quantified. The proportion of dead cells was calculated as the number of dead cells divided by the total number of cells.

### MTT cell proliferation assay

As described [14], cell lines were routinely passaged when reaching mid-log phase. For the determination of growth rate inhibition, cells were plated in triplicate in 96-well plates at a density of 4,000 cells per well in 100 μL of cell culture medium. The cells were cultivated for 15~24 h to reach a cellular confluence of 20~30% before being exposed to a test compound in a concentration range from 0.1 nM to 10 μM in a 3-fold serial dilution, 0.5 nM to 1 μM in a 2-fold of serial dilution or 1 nM to 2 μM in a 2-fold of serial dilution. Cells exposed to the solvent (0.1% of DMSO) were used as a negative control. At the end of a 3-day treatment, 20 μL of MTT (stock solution 5 mg/mL) was added to each well before incubation at 37°C for an additional 3~4 h. Next, the medium was aspirated with a BioTek ELX50 microplate washer and 50 μL of DMSO was added to each well. After shaking for 10 min, the absorbance at 570 nM was quantified with a BioTek ELX808iu microplate reader. The relative cell number was calculated as (A_570_Compound/A_570_DMSO) ×100%, with the absorbance in the control DMSO group set at 100%. For the calculation of the half-maximal inhibitory concentration (IC_50_), the concentration that causes 50% of inhibition of proliferation compared with cells treated with DMSO, the area over the curve (AOC), and E_max_ (100%—the percentage of the cell number in the treatment group relative to the DMSO control group at the highest drug concentration), the cell proliferation data were fit to the four parameters logistic curve using GraphPad Prism 8.0.2. Statistical analysis was performed using the same software. Data are presented as mean ± SD.

### Time-lapse video recording analysis of cell division

For live-cell imaging, cells were seeded into a 12-well plate and treated with LXY18 at concentrations specified in figure legends or with 0.01% DMSO as vehicle control. Bright-field images were captured every 10 min. for a total of 78 hours using an EVOS Auto FL system equipped with an onstage incubator (ThermoFisher). Mitotic cells were identified by their distinct morphological features, such as detached and rounded morphology, condensed chromosomes, partial alignment of chromosomes at the metaphase plate and cortex contraction, all of which were all readily discernible through time-lapse microscopy. The mitotic index in Fig 4A was determined by dividing the number of cells in mitosis by the total cell number in multiple randomly chosen fields. The duration of each cell spent in mitosis (Fig 4B) was also documented. The fate of cells at the endpoint of this experiment was monitored and quantified in Fig 4C.

### Immunofluorescence

Cells were cultured on glass cover slips, fixed in 4% PFA, and permeabilized with 0.1% Triton X-100 before binding of antibodies recognizing AURKB (Absin, Cat.NO. 131460) and/or human CREST autoimmune serum [14]. After staining with an appropriate secondary antibody, cells were imaged under an EVOS FL auto microscope. Secondary antibodies were Rhodamine Red^TM^-X-conjugated AffiniPure Donkey Anti-Human IgG(H+L) (Jackson, 709-295-149), Fluorescein (FITC)-conjugated Affinipure Goat Anti-Rabbit IgG(H+L) (Jackson, Cat.NO. 111-095-003).

### Western blot analysis

RIPA buffer (50mM Tris, 150mM NaCl, 1% TritonX-100, 1% sodium deoxycholate, 0.1%SDS) supplemented with a cocktail of phosphatase (Beyotime, #P1082) and protease (Beyotime, # P1005) inhibitors was used to lyse cells on ice for 15 min in 1.5 ml Eppendorf tubes. The cells were then sonicated for 10 seconds before centrifugation at 12,000 rpm for 15 min. Protein concentration was quantified using a BCA assay kit (Sangon Biotech, Cat. No. C503051-0500) according to the manufacturer’s instructions. After boiling in SDS-PAGE sample loading buffer, ~80 μg of protein was loaded into each lane of a precast protein gel (Invitrogen, NP0336BOX or NP0321BOX) and separated under a constant voltage of 100-130V for 1~2 hours. Protein was transferred to a PVDF membrane (0.22 μm) for 80 min at a constant voltage of 70V. The membrane was blocked at room temperature for 1.5 hours with 5% non-fat dry milk (Sangon, A600669-0250) suspended in TBST buffer. Primary antibody incubation was overnight at 4°C in an Odyssey blocking buffer containing 0.2% Tween 20 or alternatively in PBS containing 0.2% Tween 20. All primary antibodies were used at 1: 1000 dilution. The membrane was incubated with secondary antibody at room temperature for 1 h. An IRDye® 800CW Goat anti-Mouse IgG (Cat. #. 926–32210, Lot#C91210-09) and an IRDye® 680RD Goat anti-Rabbit antibody (926–68071, Lot#D00115-06) were used at a 1:10000 and 1:5000 dilution, respectively. Images were captured using an Odyssey CLx Imaging System and processed with Image Studio Ver 5.2.

### Kinome assay

The assay was performed with KinaseProfiler^TM^ Technology by the Eurofins Discovery Products Company. The technology utilizes a radiometric assay platform to measure catalytic activity directly, enabling the detection of ATP-competitive, substrate-competitive, and allosteric inhibitors [18]. The assay involved incubating 430 different kinases with a buffer composition specified in Eurofins’s KinaseProfiler™ Service Assay Protocols v87. The test compound was prepared in 100% DMSO at a 50x final assay concentration. The compound was added to the assay well as the first component, followed by the remaining components. In the standard KinaseProfiler service, there was no pre-incubation step between the compound and the kinase before the reaction was initiated. The reaction was initiated by the addition of the Mg/ATP mix and lasted for 120 min at room temperature before adding phosphoric acid to a final concentration of 0.5%. An aliquot of the terminated reaction was spotted onto a filter and washed four times in 0.425% phosphoric acid and once in methanol before drying and scintillation counting.

### Motor activity assay

The motor activity assays were performed by the Cytoskeleton Company with the KINESIN ELIPA BIOCHEM KIT (Cytoskeleton, Cat. No. BK060).

#### a. MKLP2’s ATPase activity assay

A test compound was prepared at 100x concentration in DMSO and used in a seven-point concentration response assay with final concentrations at 20, 6.7, 2.2, 0.74, 0.25, 0.8, and 0.03 μM. A motor mix was prepared by mixing the following reagents sequentially at RT, including 6.4 mL of 15 mM Pipes-NaOH pH 7.0, 10 mM MgCl_2_, 20 μM Tx (Buffer 1), 3 mL of 5x MESG (ELIPA 1 reagent, Cat. # BK051), 0.64 mL 2.5 mg/mL MTs (3 x 10mg Cat. # MT002-XL resuspended in 12 ml of Buffer 1), 0.25 mL 1 μg/μL MKLP2 protein, and 0.15 mL 100 x PNP (ELIPA 2 reagent, cat. # BK051). 70 μL of the motor mix was dispensed into each well of a 96-well microplate, followed by the addition of 35 μL of 3 mM ATP to initiate the reaction. After 5-second rapid circular mixing, OD values at 360 nm wavelength were recorded at 22°C for 31 times with a 40-second interval between each recording.

#### b. Motor panel screening

Dilute compounds into DMSO at 100x concentration. Pipette 1 μL of DMSO solution directly into each well. Sequentially mix the following reagents at room temperature to obtain the “motor mix”: 2 mL 15 mM Pipes-NaOH pH 7.0, 10 mM MgCl_2_, 20 μM Taxol (Buffer 1), 1 mL 5x MESG (ELIPA 1 reagent, Cat. # BK051), 0.2mL (Plate 1) or 1 mL (Plate 2 & 3) of 2.5 mg/mL MTs (1 x 10mg Cat. # MT002-XL resuspended in 4 mL of Buffer 1), and 0.05 mL 100 x PNP (ELIPA 2 reagent, cat. # BK051). To initiate the reaction, there was 70 μL of the motor mix used in each well and 35 ul of 3 mM GTP used for KIF7 and 35 μL of 3 mM ATP used for the other motor proteins. The OD_360_ was recorded as described above. Information related to the motor panel screening assay is given in S3 Table.

## Results

### Cancer cell lines refractory to AURKB inhibitors are sensitive to LXY18

**Fig 1:**
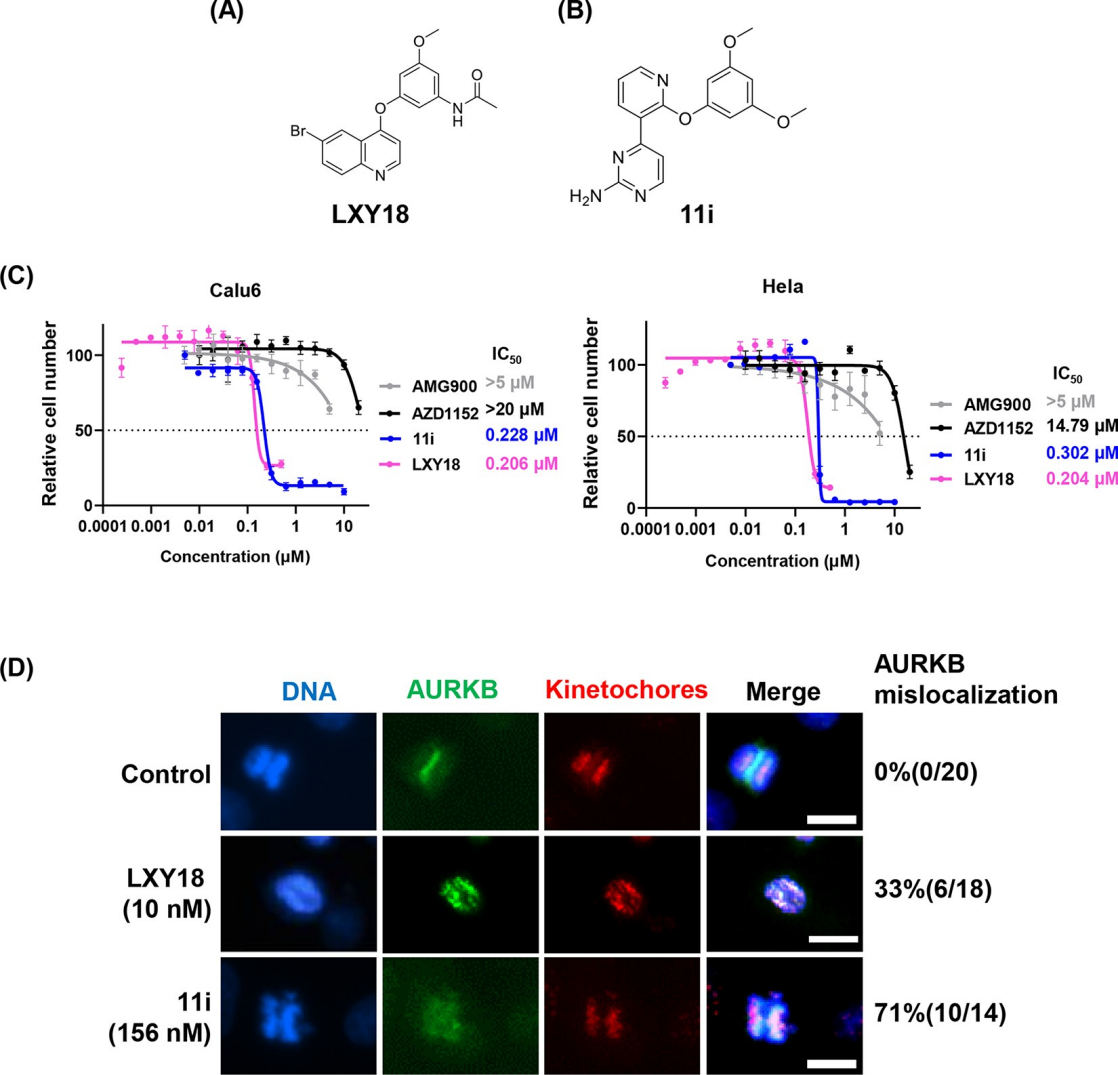
The structure and bioactivity of LXY18 and 11i. (A, B) The chemical structures of LXY18 and 11i. (C) Concentration-response curves for Calu-6 and Hela cells as determined by the MTT assay. Cell numbers are relative to vehicle control (0.1% DMSO). Each point represents the mean ± SD from three independent experiments with measurements done triplicate. The IC_50_ was calculated with GraphPad Prism 8.0.2. (D) The localization of AURKB. RPE-MYC^Bcl2^ cells were treated for 6 hours with the indicated compounds before immunofluorescent staining for AURKB and kinetochores with human CREST serum. DNA staining was with DAPI. Cells treated with vehicle (0.1% of DMSO) were used as a negative control. The numbers of early anaphase cells scored and those with AURKB mislocation under each treatment are indicated. Bar: 10μm.

To investigate if there was similar sensitivity between LXY18 and AURKB catalytic inhibitors, we utilized the human cervical cancer cell line, Hela, and the lung adenocarcinoma cell line, Calu-6 (Fig 1A and 1C), both of which respond poorly to AURKB small molecule inhibitors. An MTT assay, which measures the metabolic activity of viable cells, was conducted. For the Hela and Calu-6 lines, the AURKB inhibitors AMG900 and AZD1152 both failed to reach a point where viable cell number was half the maximal number of control-treated cells (Fig 1C). This was the case despite their low nanomolar potency in inhibiting AURKB kinase activity [19, 20]. In contrast to catalytic inhibitors of AURKB, LXY18 effectively suppressed viability with a half-maximal concentration (IC_50_) under 206 nM in both cell lines (Fig 1C). We also assayed a second AURKB relocation blocker, named compound 11i [21], which is structurally distinct from LXY18 (Fig 1B). 11i also displayed a nanomolar IC_50_ of 228~302 nM in the MTT assays, comparable to that seen with LXY18 (Fig 1C).

Consistent with our recent report [19], LXY18 and 11i both induced the mis-localization of AURKB during mitosis in the RPE-MYC^Bcl2^ cell line (Fig 1D). Normally, AURKB is still detected in the region of the metaphase plate between separating chromosomes during early anaphase (Fig 1D). However, when treated with LXY18, 33% of early anaphase cells lacked this localized AURKB staining. Instead, AURKB staining was still present on chromosomes or present as a smear between segregating chromosomes. When treated with 11i, 71% of anaphase cells suffered these defects in AURKB localization. Collectively, these findings demonstrate that LXY18, along with other AURKB relocation blockers, induce cytotoxic effects distinct from AURKB catalytic inhibitors. They are cytotoxic even in cell lines poorly responsive to AURKB small molecule inhibitors.

### LXY18 exerts broad-spectrum activity against various cancer cell lines

To investigate whether LXY18 displayed a similar pattern in these cells or harbors distinct, unknown limitations, we examined the cytotoxicity of LXY18 in a panel of 18 human cancer cell lines, including some cell lines poorly responsive to AURKB inhibitors, which we uncovered using the cancer cell line Dependency Map Portal (DepMap; https://depmap.org/portal/) (S1 Table). The panel represented cancer cells derived from diverse organs, including breast, ovary, prostate, colon, lung, stomach, and skin (S1 Table). Collectively, these cell lines harbor a broad spectrum of oncogenic driver mutations (S2 Table) that promote tumorigenesis and drug resistance [22].

**Fig 2:**
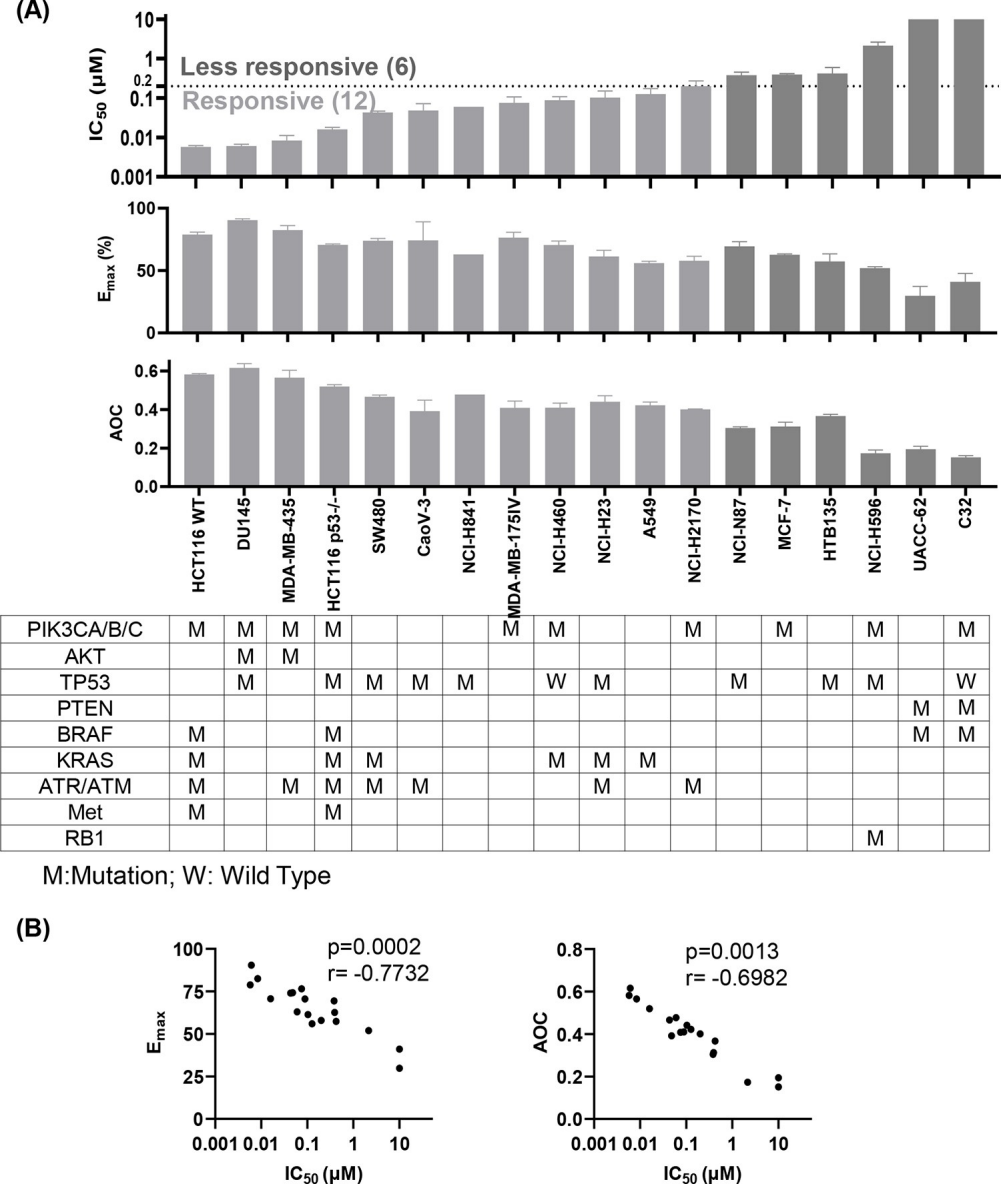
LXY18 exerts broad-spectrum activity in suppressing the growth of cancer cell lines. (A) The IC_50_, E_max_, and AOC of LXY18 in 18 cancer cell lines treated over a wide concentration range for 3 days before viable cell number was quantified by MTT assay. The IC_50_, E_max_, and AOC were calculated with GraphPad Prism 8.0.2. Histograms show the combined data from three independent experiments presented as mean ± SD. (B) The reverse correlation between IC_50_ and either E_max_ or AOC.

Treatment with LXY18 exerted variable effects (Fig 2 and S1 Fig). Among the different cell lines, concentration-response curves varied dramatically with heterogeneity in the slope (h), maximal effect at the highest drug concentration (E_max_), IC_50_, and the area over the curve (AOC). AOC serves as a descriptor that combines the potency and efficacy into a single parameter. In light of the heterogeneity of response, we divided the 18 cell lines into 12 responsive lines and 6 less-responsive lines with a cutoff of IC_50_ of 0.2 μM (Fig 2A). IC_50_ values were inversely correlated with either AOC or E_max_ (Fig 2B). Deletion of tumor suppressor gene TP53 was the most common driver mutation, yet neither the IC_50_ nor E_max_ of cell lines treated with LXY18 were correlated with the status of TP53 (Fig 2A). Cancer cell lines with amplification of MET (HCT116), activating mutations in RAS family members (HCT116, SW480, NCI-H460, NCI-H23 and A549) and PI3K subunits (HCT116, DU145, MDA-MB-435, NCI-H460 and NCI-H2170) were all responsive to LXY18, indicating that these cancer drivers do not correlate absolutely with resistance to LXY18. Seven out of the twelve LXY18-responsive cell lines were poorly responsive to the AURKB inhibitors (Fig 2A and S1 Table), consistent with our findings in Hela and Calu-6 cells (Fig 1).

#### LXY18 elicits apoptosis in cancer cells but not in non-transformed cells

**Fig 3:**
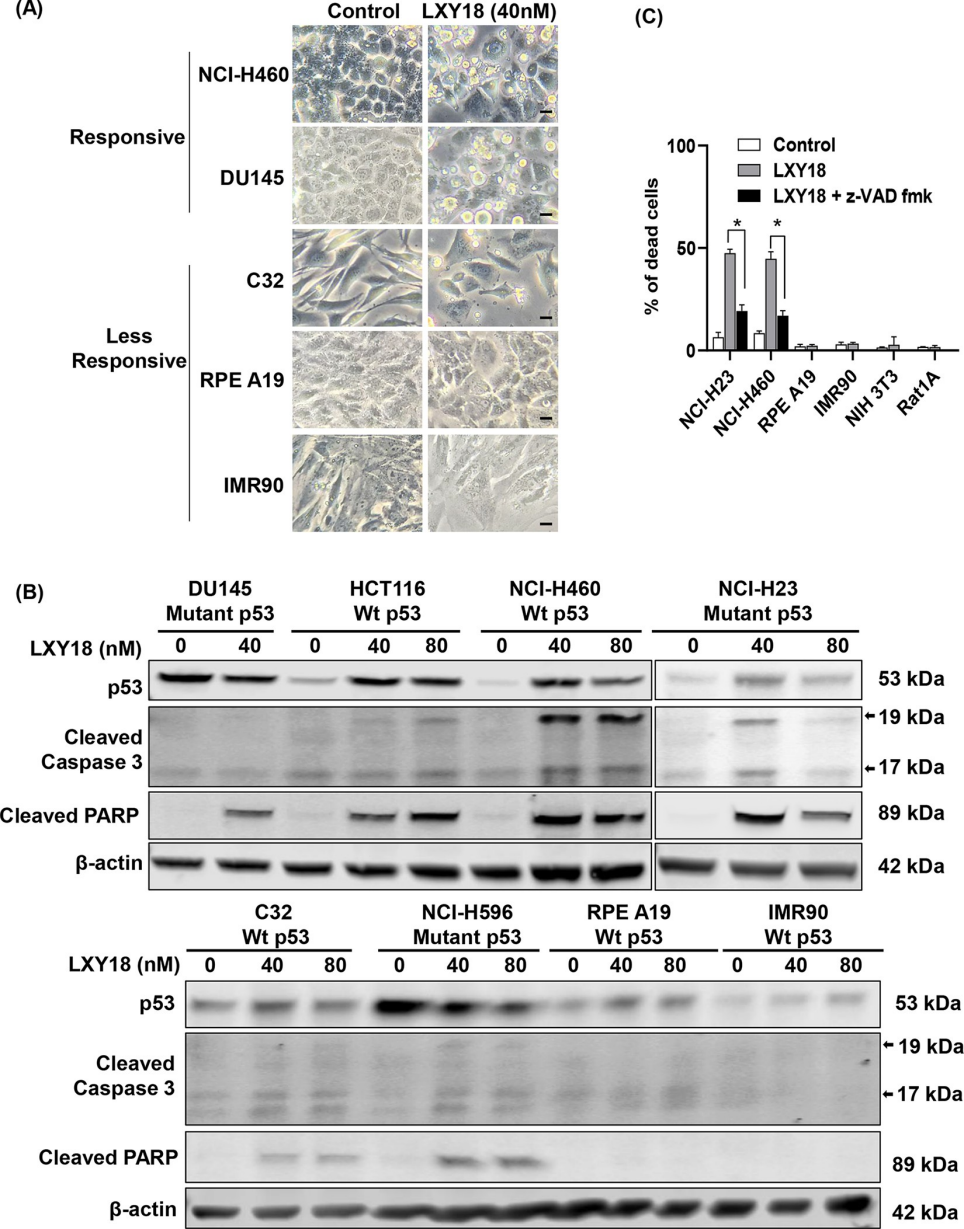
LXY18 elicits apoptosis in cancer lines but not in nontransformed cell lines. (A) Cell morphology in response to LXY18. The indicated cell lines were exposed to 40 nM of LXY18 for 48 hours. Images were acquired using an inverted tissue culture microscope. Scale bar, 10 μm. (B) LXY18 elicits apoptosis in cancer cell lines. The indicated cell lines were exposed to 40 or 80 nM of LXY18 for 48 hours prior to lysis and Western analysis of protein. (C) Cell death could be rescued by inhibiting caspases. NCI-H23 and NCI-H460 were treated for 48 hours with 40 nM LXY18 either alone or in combination with 100 μM of Z-VAD fmk for 24 hours. RPE A19 and IMR90 cell lines were also treated with 40 nM of LXY18 for 48 hours, while NIH 3T3 and Rat1A cell lines were exposed to 40 nM of LXY18 for 72 hours. Vehicle control was 0.1% DMSO. At the endpoint, the Trypan blue exclusion assay was used to detect dead/living cells and the percent dead cells in the population was calculated. Data from three independent experiments with triplicate measurements are presented as mean ± SD. *p<0.0001.

Substantial floating dead cells were observed with the majority of cell lines exposed to LXY18. Cell death was very pronounced in DU145 and NCI-H460 cells after treatment with LXY18 (Fig 3A). In contrast, cell death was mild in the cancer cell line C32 and absent in the non-transformed human retinal pigment epithelial cell line, RPE A19, and the non-transformed human lung fibroblast line, IMR90. Four responsive cell lines (HCT116, DU145, NCI-H23 and NCI-H460), and two less-responsive cell lines (C32 and NCI-H596) were chosen to examine apoptosis markers. LXY18 treatment elevated cleavage of poly (ADP-ribose) polymerase (PARP) and cleavage of caspase 3 in all cancer cell lines examined. Higher levels of cleaved PARP and cleaved caspase 3 were induced by LXY18 in responsive cell lines relative to the less-responsive cell lines (Fig 3B and S2 Fig). LXY18-treated cancer cells could be rescued by a pan-caspase inhibitor, carbobenzoxy-valyl-alanyl-aspartyl-[O-methyl]- fluoromethylketone (z-VAD-fmk), which reduced cell death by 2.47-fold and 2.63-fold in NCI-H23 and NCI-H460 cell lines, respectively (Fig 3C). The rescue experiment confirmed that the lethal effect elicited by LXY18 could be primarily attributed to apoptosis. In contrast, neither Trypan blue positive dead cells nor apoptosis markers were detected in non-transformed RPE A19 and IMR90 (Fig 3A and 3B). Trypan blue exclusion assays also failed to detect the presence of dead cells in RPE A19, IMR90, mouse embryo fibroblasts NIH-3T3, or rat embryo fibroblasts rat1A cells after treatment with LXY18 (Fig 3C). Thus, LXY18 elicits cell death preferentially in cancer cells as opposed to non-transformed cells.

Chemotherapeutics are known to induce p53-mediated apoptosis in cancer cells and mutations in p53 often cause drug resistance [23, 24]. Robust induction of p53 by LXY18 was detected in two responsive cell lines HCT116 and NCI-H460, both of which harbor wild-type p53 (Fig 3B). p53-mutant cell lines DU-145, NCI-H596, and NCI-H23 lacked or had only modest induction of p53 in response to LXY18. Likewise, a less-responsive cancer cell line C32 and the non-transformed cell lines RPE A19 and IMR90 lacked robust induction of p53 despite having wild-type *TP53*. Since LXY18 elicited robust apoptosis irrespective of *TP53* status, functional p53 protein was not required for the induction of apoptosis by LXY18.

### LXY18-triggered lethality occurs mainly during mitosis

We conducted time-lapse imaging experiments to identify the stage of the cell cycle in which LXY18-treated cells succumbed to apoptosis. This method also enabled us to examine the fate of the minority of cells that did successfully transition the cell cycle. In response to LXY18 the mitotic index (MI) was elevated.

**Fig 4:**
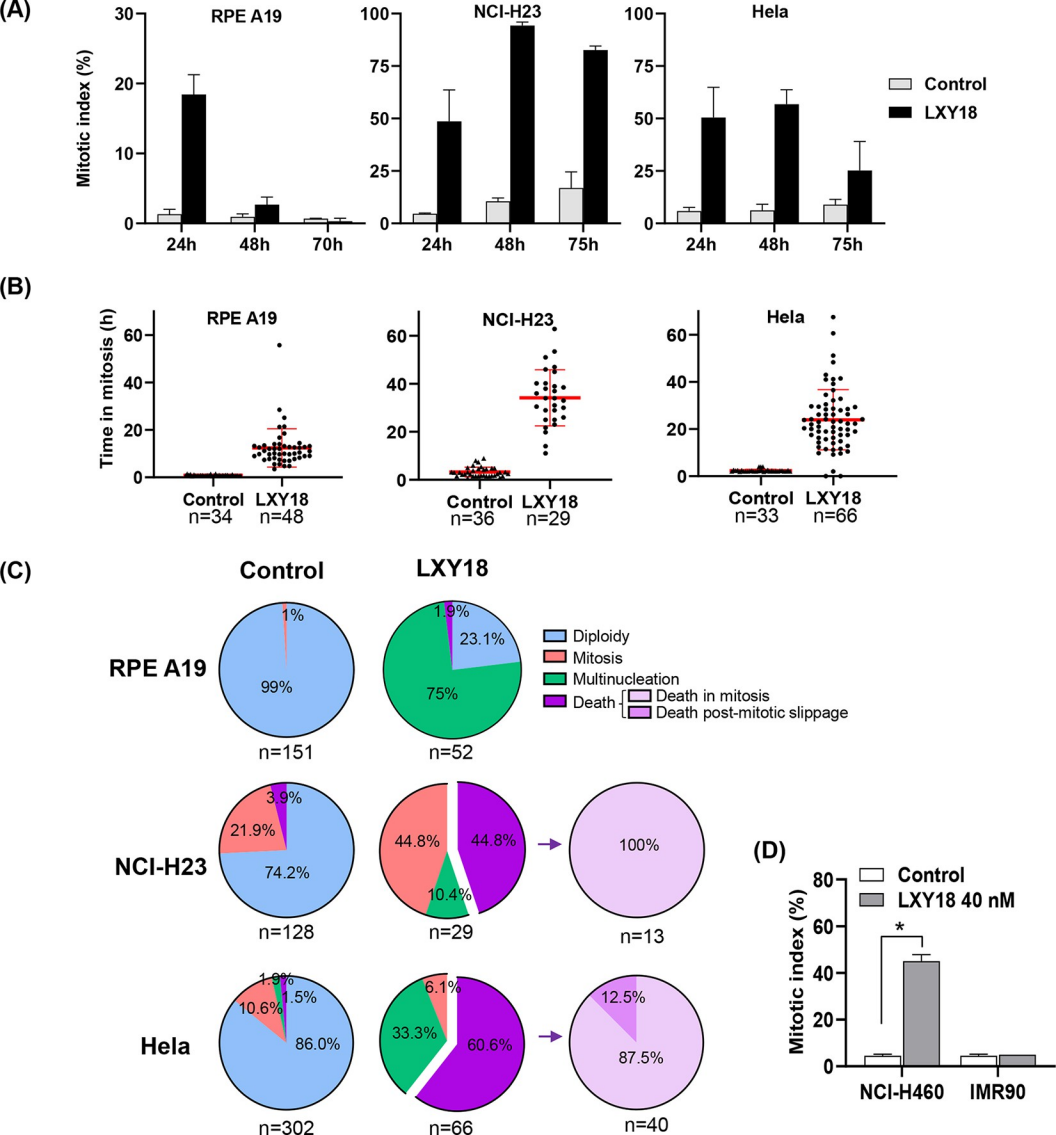
LXY18 elicits a prolonged mitotic arrest, cytokinetic failure, and cell death. Cells were subjected to a time-lapse analysis following exposure to 40nM LXY18 with a duration of 70 hours (RPE A19) or 75 hours (NCI-H23 and Hela). (A) The histogram shows the mitotic index at 24, 48, and 72 hours. A total of 200–600 cells were scored for each data point. (B) The duration of mitotic arrest was quantified for each cell line with LXY18 treatment (n is the number of mitotic cells scored in each group). (C) Endpoint analysis showing the fate of cells followed throughout the entire time course of the experiment. (D) Histogram showing the mitotic index as mean ± SD (*p<0.0001). Cells were treated with 40 nM LXY18 for 48 hours or with vehicle control (0.1% DMSO).

Elevated MI occurred in RPE-A19, Hela, and NCI-H23 cells, albeit to a variable extent and duration (Fig 4A). The MI in RPE-A19 cells reached a peak of 18.4% at 24 hours, and declined to 2.7% after 48 hours, only to fade to background levels after 72 hours. In contrast, elevation of MI persisted in both cancer cell lines. The MI in NCI-H23 and Hela cells reached a peak after 48 hours (94.3% and 56.8% respectively), and stayed elevated at 72 hours (82.6% and 25.2%) (Fig 4A). The MI was also persistently elevated in LXY18-responsive NCI-H460 cancer cells but not in non-transformed IMR90 (Fig 4D).

**Fig 5:**
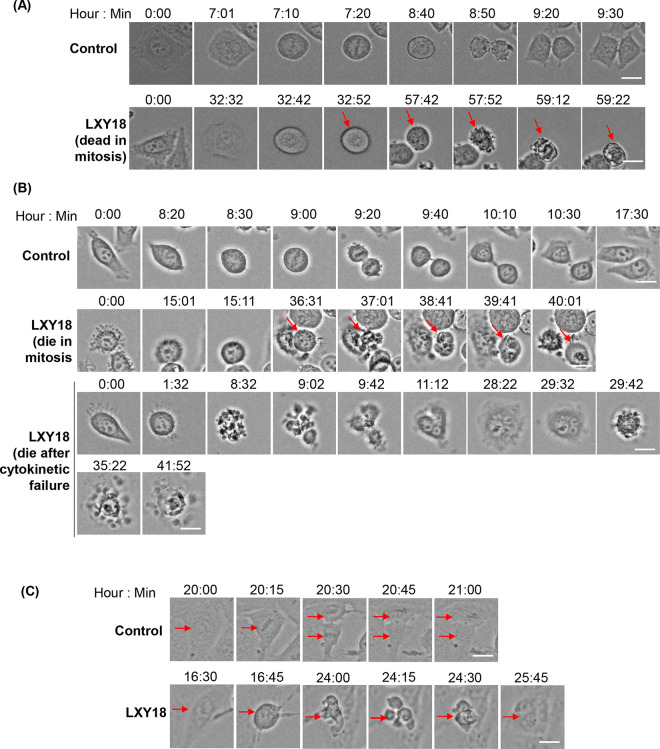
Time-lapse images of cells treated with LXY18. Cells were treated with or without 40 nM of LXY18 and then subjected to time-lapse video filming. Representative images are presented. (A) NCI-H23. (B) Hela. (C) RPE A19. Bars: 10 μm.

In the presence of LXY18, the RPE-A19, Hela and NCI-H23 cells spent 12.4, 23.9, and 34.1 hours in mitosis, compared to 0.9, 2.4, and 3.2 hours, respectively, without drug treatment (Fig 4B). Extensive mitotic cell death occurred in NCI-H23 (44.8%) and Hela (60.6%) cells (Figs 4C, 5A, 5B). However, a small fraction escaped from death and formed polyploid cells. Through the time-lapse imaging experiment, we not only observed that multinucleated cells resulted from a failure of cytokinesis, but also observed that some of these polyploid cells also succumbed to cell death (Figs 4C and 5B), revealing a secondary wave of lethality.

In contrast with the findings in cancer cells, LXY18-induced death was not observed in time-lapse imaging of RPE-A19 cells (Figs 4C and 5C), consistent with previous observations (Fig 3). Unlike cancer cells, RPE-A19 and IMR90 cells experienced only a transient mitotic arrest with LXY18 (Fig 4A, 4B and 4D). We speculate that this shorter mitotic arrest might allow non-transformed cells to avoid death as a prolonged arrest in mitosis is typically required to trigger mitotic catastrophe [25]. When LXY18 was present, some RPE-A19 cells underwent an unsuccessful multipolar division. The ingression of cortex subsequently reversed, enabling the cells to revert to interphase while displaying multinucleation (Fig 5C).

Collectively, these findings indicate that LXY18 provoked a biphasic cell death selectively in cancer cells. One fraction of cells underwent apoptosis in mitosis after a prolonged mitotic arrest whereas another fraction of cells died after a mitotic slippage or after undergoing a failure of cytokinesis. Non-transformed cells did not die in response to LXY18. The genomic or cellular determinants that render cancer cells, but not non-transformed cells, sensitive to the lethal effect of LXY18 is an important area for future investigation.

### S phase arrest protects cells from LXY18-induced death

We observed an LXY18-induced relocation failure of the CPP complex which culminated in mitotic catastrophe for the majority of cells. Additionally, cells that did manage to progress through mitosis were susceptible to post-mitotic death signaling. This suggests that arresting cycling cells prior to mitotic entry could protect from LXY18-induced lethality.

**Fig 6:**
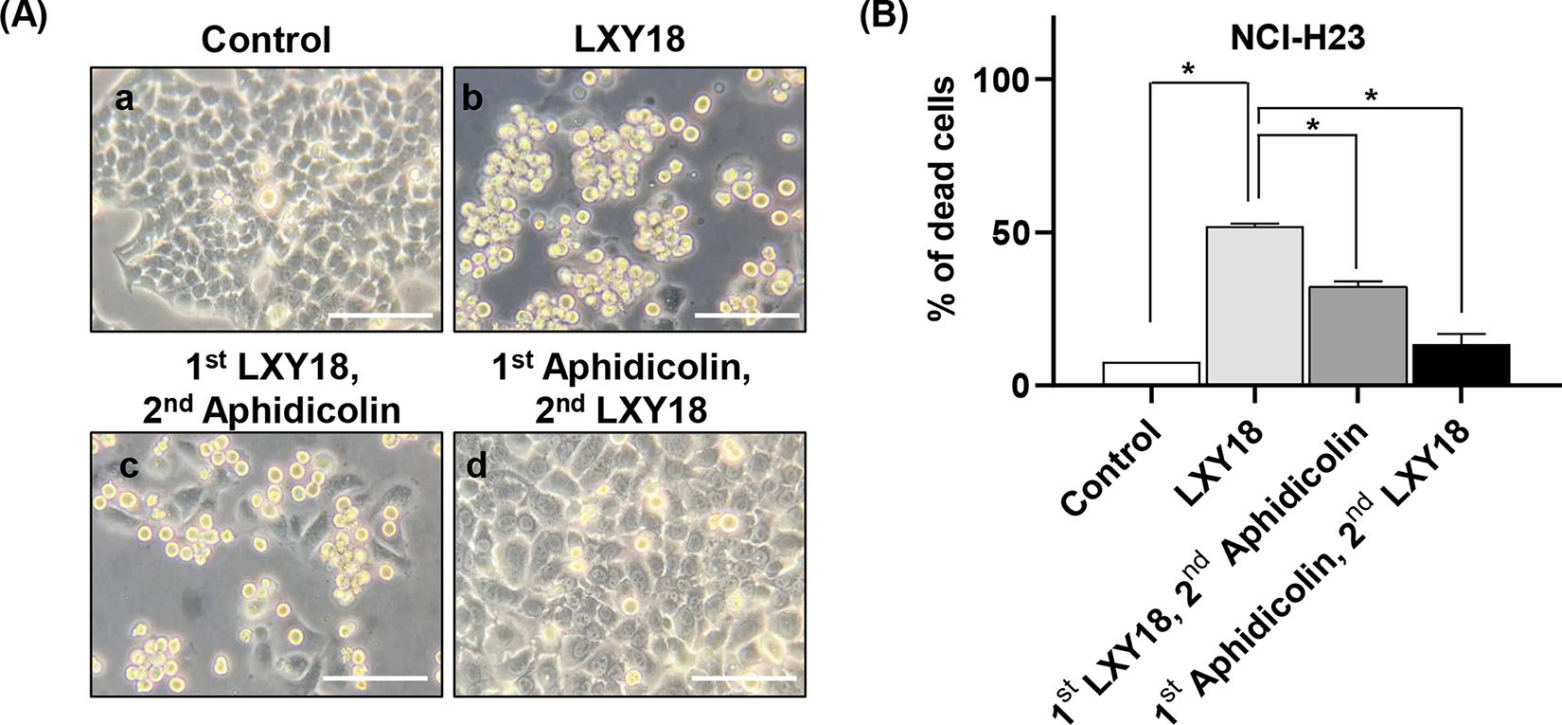
Aphidicolin protects cells from cell death elicited by LXY18. NCI-H23 cells were treated as indicated. (A) The morphology of NCl-H23 cells. Scale bars, 100 μm. (a) Vehicle control (0.1% DMSO) for 48 hours. (b) LXY18, 40 nM for 48 hours. (c) Results of assay with 1^st^ LXY18 (40nM) + 2^nd^ Aphidicolin (3 μg/mL). LXY18 was for 48 hours with addition of 3 μg/mL aphidicolin for the last 24 hours. (d) Results of assay with 1^st^ Aphidicolin (3 μg/mL) + 2^nd^ LXY18 (40nM). LXY18 was for 48 hours, with aphidicolin given 24 hours before the addition of LXY18. Cell viability was determined by Trypan blue assays. (B) The percentage of dead cells. The percentage of dead cells in 6A was quantified. Combined data from three independent experiments with triplicate measurements are presented as mean ± SD. *p<0.0001.

To test this hypothesis, we treated NCI-H23 cells with aphidicolin, a DNA synthesis inhibitor that blocks the cell cycle at early S phase. In our experiments, we exposed cells to aphidicolin (3 μg/mL) for 24 hours, which effectively diminished BrdU incorporation [26]. In separate assays, we exposed cells to LXY18 either prior to or following the exposure to LXY18. When aphidicolin was added before LXY18, no cell death or multinucleation of cells took place (Fig 6A and 6B). As expected, treatment with LXY18 alone induced lethality (52% of NCI-H23 cells), supporting the hypothesis that entry into mitosis makes cancer cells susceptible to LXY18-induced death.

The sequential treatment of cells was then reversed so that NCI-H23 cells were exposed to LXY18 for 24 hours before aphidicolin. By the time aphidicolin was added, a fraction of the cells had already undergone a cytokinetic failure, while many cells were arrested in mitosis. This second treatment strategy still conferred some protection, reducing the percentage of dead cells from 52% to 32% (Fig 6A and 6B). It was noted that more multinucleated cells accumulated with the combination treatment compared with cells treated with LXY18 alone (Fig 6A). Collectively, these data suggest that entry into mitosis is essential for LXY18 to elicit lethality.

### LXY18 fails to inhibit the catalytic activities of MKLP2 and NEKs 6, 7 and 9

Our time-lapse video recording study confirmed that treatment-induced multinucleation was due to a failure in cytokinesis, a phenotype consistent with the inhibition of proper AURKB localization during mitosis [14]. To identify a possible target protein for LXY18 that could be responsible, we tested known regulators of AURKB relocation including NEK6, NEK7, and NEK9, and the motor kinesin-like protein MKLP2.

**Fig 7:**
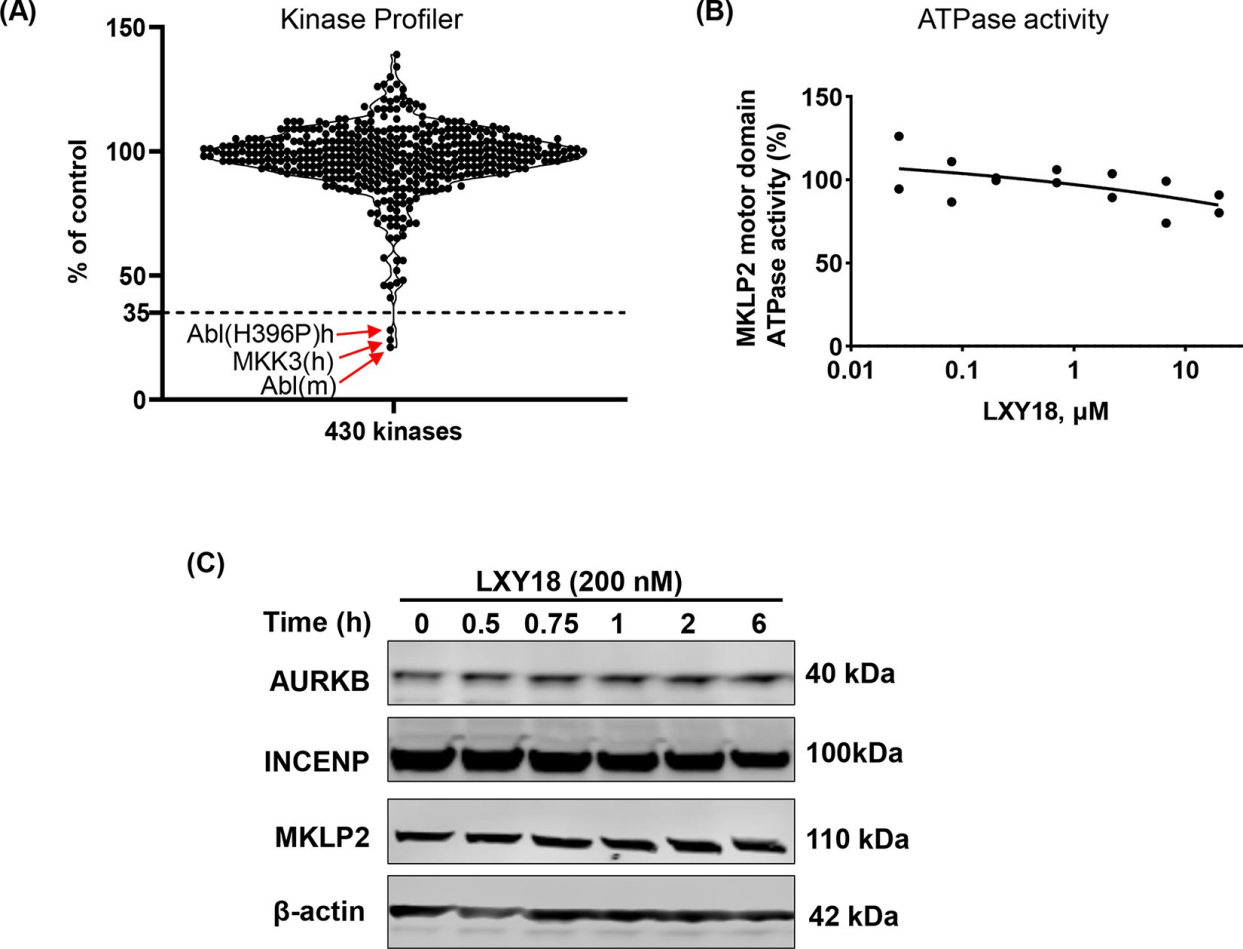
LXY18 fails to inhibit the catalytic activities of MKLP2 and NEK6, NEK7, and NEK 9. (A) Kinome-wide selectivity profiling of 430 kinases with LXY18. The scatter plot shows the percentage of inhibition of each of 430 kinases by 2 μM of LXY18. (B) The impact of LXY18 on the MKLP2’s ATPase activity. (C) Western blot for the expression of AURKB, INCENP, and MKLP2 after treatment of NCI-H23 cells for the indicated time with 200 nM of LXY18.

A kinome assay with a panel of 430 kinases, including NEK proteins was performed. This experiment also allowed us to assess the overall impact of LXY18 on the human kinome. LXY18 did not inhibit the kinase activities of NEK6, NEK7 or NEK9 at a concentration of 2 μM, which was much higher than the minimum effective concentration (MEC) of 10 nM at which LXY18 mislocalizes AURKB [15]. Consistent with the findings in cell-based assays [15], the catalytic activities of AURKA and AURKB were not affected in the kinase panel. The only positive hits in the kinome assay were MKK3, ABL, and an activating mutant ABL (H396P) (Fig 7A). However, inhibition of none of these kinases could block the relocation of AURKB at the anaphase onset. These findings indicate that LXY18 selectively mislocalizes AURKB without affecting the activities of nearly all kinases examined, consistent with previous findings from LXY18 analogs [14].

We then examined if LXY18 could inhibit the ATPase of MKLP2 and other mitotic motor proteins. LXY18 failed to inhibit the microtubule-stimulated ATPase activity of MKLP2 even at a high concentration of 20 μM (Fig 7B and S4A Fig). In addition, the expression levels of MKLP2, AURKB, and INCENP remained unchanged in the presence of LXY18 (Fig 7C and S3 Fig). At a concentration of 20 μM, LXY18 exerted only 20–30% inhibition of ATPase activity of KIFC3, KIF3C, MCAK, and Chromokinesin. Inhibition of KHC, CENP-E, EG5/KSP/KIF11, MKLP1, KIF7 and KIF22 was less than 20% (S4B and S4C Fig). These data provide no evidence that the mislocalization of AURKB by LXY18 could be attributed to the inhibition of any of the kinases and motor proteins. The exact mechanism by which LXY18 targets the localization of the CPP complex and its associated AURKB activity remain under investigation.

## Discussion

### LXY18 elicits prolonged mitotic arrest and apoptosis selectively in cancer cells

LXY18 is a potent, and selective quinoline compound that disables the mitotic function of AURKB by preventing its proper localization during mitosis, not by inhibiting its catalytic activity [15]. In this study, we demonstrated that LXY18 attacks cancer cells by triggering mitotic arrest and apoptosis, presumably following prolonged activation of the spindle checkpoint response. The few remaining cells escaped from mitotic death, perhaps through a mitotic slippage whereby the spindle checkpoint response was bypassed, and ultimately perished in the next interphase. This post-mitotic death was, at least partially driven by illegitimate DNA synthesis. Our investigation also demonstrated that cells arrested in early S-phase were immune to apoptosis induction by LXY18, consistent with the assumption that LXY18 attacks the mitotic machinery.

There are two unique features associated with the antimitotic agent LXY18. Firstly, it triggers prolonged mitotic arrest and apoptosis in cancer cell lines but not in dividing non-transformed cells. The mechanism underpinning the cancer cell-selective effect is enigmatic. Disabling AURKB by RNAi or catalytic inhibitors is known to push cells out of mitosis rather than blocking mitotic progression [12]. Since the localization and kinase activity of AURKB in early mitosis were normal in cells treated with LXY18, it is unlikely that the kinetochore function of AURKB in maintaining the spindle checkpoint response was compromised. One might speculate that LXY18 disables an upstream regulator that specifies AURKB localization and its disruption by LXY18 led to a persistent activation of the spindle assembly checkpoint response in tumor cells, but not in non-transformed cells. Identifying the target and understanding the precise mechanism underlying this cancer cell-selective mitotic arrest is an important research area to develop and will enhance our understanding of how to use LXY18 as an oncology drug.

A second unique feature is that cancer cells refractory to AURK catalytic inhibitors were still highly sensitive to LXY18. This sensitivity suggests that blocking the relocation of AURKB may be more efficient for inducing apoptosis than inhibiting its catalytic activity. Alternatively, the increased sensitivity of cancer cells to LXY18 could be attributed to the inhibition of an upstream regulator of AURKB which has additional roles to play in mitosis, aside from regulating AURKB localization. It is also possible that inhibition of an upstream regulator of AURKB relocation led to a prolonged mitotic arrest that enables the spontaneous apoptosis. Further investigation to distinguish these possibilities will provide important details about how LXY18 and related drugs target the mitotic apparatus to produce a potent anticancer effect.

### LXY18 prevents the relocation of the CPP complex without inhibiting MKLP2 and NEK6,7,9

Our findings do provide some important evidence regarding what is not targeted by LXY18. This compound does not inhibit the ATPase activity of recombinant MKLP2, nor did it inhibit the catalytic activity of NEK6, NEK7 or NEK9. These findings do not exclude the possibility that LXY18 might disable one of these AURKB relocation regulators independently of inhibiting their catalytic activities. For example, it may be important to investigate whether LXY18 can inhibit the function of MKLP2 in both the binding and bundling of microtubules, as well as processivity of its movement along microtubules. Other regulators of AURKB relocation also need to be investigated as potential LXY18 targets in the future. Examples are the MPS1-binder-related protein 1 (Mob1) [27], cyclin-dependent kinase 1 (CDK1) [28], spindle checkpoint kinase mitotic arrest deficient (Mad2) [29], Cullin-RING E3 ubiquitin ligase (E3 ligase Cul3) and its adaptors, Kelch-like proteins (KLHL9) and (KLHL13) [30], the infraglabellar transport (IFT) machinery components [31], the Wild-Type p53-Induced Phosphatase 1 (WIP1) [32], and the microtubule-binding protein regulator of cytokinesis 1 (PRC1) [33].

### Blocking AURKB relocation as a novel approach in oncology treatment

The mechanism of action of LXY18, whereby AURKB relocation is inhibited in mitotic cells may also be an effective strategy to avoid the development of drug resistance. LXY18 does not appear to bind to the conserved active site of AURKB or other kinases. In addition, LXY18 and compounds with a similar mechanism of action may not be expected to affect AURKB-dependent cellular processes in non-dividing cells. LXY18 and its analogs are more effective at reducing cell viability compared to AURKB kinase inhibitors [14]. This superior cytotoxicity is likely because LXY18 acts as a mitotic blocker, rather than a mitosis driver, as has been demonstrated for inhibitors of AURKB catalytic activity. It is important to evaluate whether the more potent cytotoxicity of LXY18 translates into an improved therapeutic efficacy for LXY18 relative to AURKB inhibitors in the future. Since some oncogenic alterations such as myelocytomatosis (*MYC)* deregulation, *TP53* or retinoblastoma protein (*RB)* deletion are known to underly mitotic defects in cancer and prime cells for even further disruption of mitotic processes [34–37], interfering with the mitotic localization of AURKB might amplify these mitotic vulnerabilities better than inhibiting AURKB catalytic activity [38]. Alternatively, disabling alternate targets, perhaps upstream of AURKB in it signaling pathway, may further exacerbate mitotic defects in a manner that is not recapitulated by AURKB catalytic inhibitors. A genetic signature that can predict enhanced sensitivity to LXY18’s anticancer activity needs to be explored. This will enable the development of LXY18 or an analog as a precision cancer treatment.

## Supporting information

S1 Fig Concentration-response curves of LXY18. Each cell line was treated with LXY18 at a concentration range with 12 concentration points in a 2 or 3-fold dilution. The number of viable cells was determined by the MTT assay 72 h after the initiation of treatment.
(TIF)

S2 Fig The original blots for the display are in Fig 3B. (A) One membrane was cut into the upper part (I) and the bottom part (II). The upper part was probed for cleaved-PARP (I), and the bottom part was probed for cleaved-caspase 3 (II). (B) One whole membrane was sequentially probed for p53 (Ia) and β-actin (Ib). (C, D) One membrane was cut into the upper part (I), the middle part (II), and the bottom part (III). The upper part was probed for cleaved-PARP (I), and the middle part (II) was sequentially probed for p53 (IIa) and β-actin (IIb). The bottom part was probed for cleaved caspase 3 (III).
(TIF)

S3 Fig The original blots for the display are in Fig 7C. One membrane was cut into the upper part (I) and the bottom part (II). The upper part was sequentially probed for MKLP2 (Ia) and INCENP (Ib). The bottom part was sequentially probed for AURKB (IIa) and β-actin (IIb).
(TIF)

S4 Fig The effect of LXY18 on microtubule-stimulated ATPase activity of motor proteins. The microtubule-stimulated ATPase activity of the indicated motor proteins was determined in the presence of the indicated concentrations of LXY18. An ATP-competitive inhibitor AMPPNP at 1mM and a reaction mixture lacking motor proteins were used as positive controls for each test. DMSO, the solvent for LXY18, was used as a negative control.
(TIF)

S1 Table The IC50 of AURK inhibitors in different 11 human cancer cell lines in Depmap. IC50 data are from the genomics of drug sensitivity in cancer website (https://www.cancerrxgene.org).
(DOCX)

S2 Table Oncogenic driver mutations in 18 human cancer cell lines. The table shows 18 human cancer cell lines examined, their related cancer types, and the precise oncogenic alterations recognized in each cell line.
(DOCX)

S3 Table Motor protein assay information. The table provides information about the motor proteins used in a motor protein assay, including the amount of motor protein used per 100 uL reaction.
(DOCX)

S4 Table Reagents, antibodies, software, and equipment information. The table provides comprehensive information regarding several types of items used in the experiment, including reagents, antibodies, software, and equipment.
(DOCX)

S1 Graphical abstract (TIF)

S1 Raw images (PDF)
