# Supplementary figures and images for: The Aurora kinase B relocation blocker LXY18 triggers mitotic catastrophe selectively in malignant cells
Source: PLoS One. 2023 Oct 30;18(10):e0293283. doi: 10.1371/journal.pone.0293283 (PMC10615259; doi:10.1371/journal.pone.0293283)

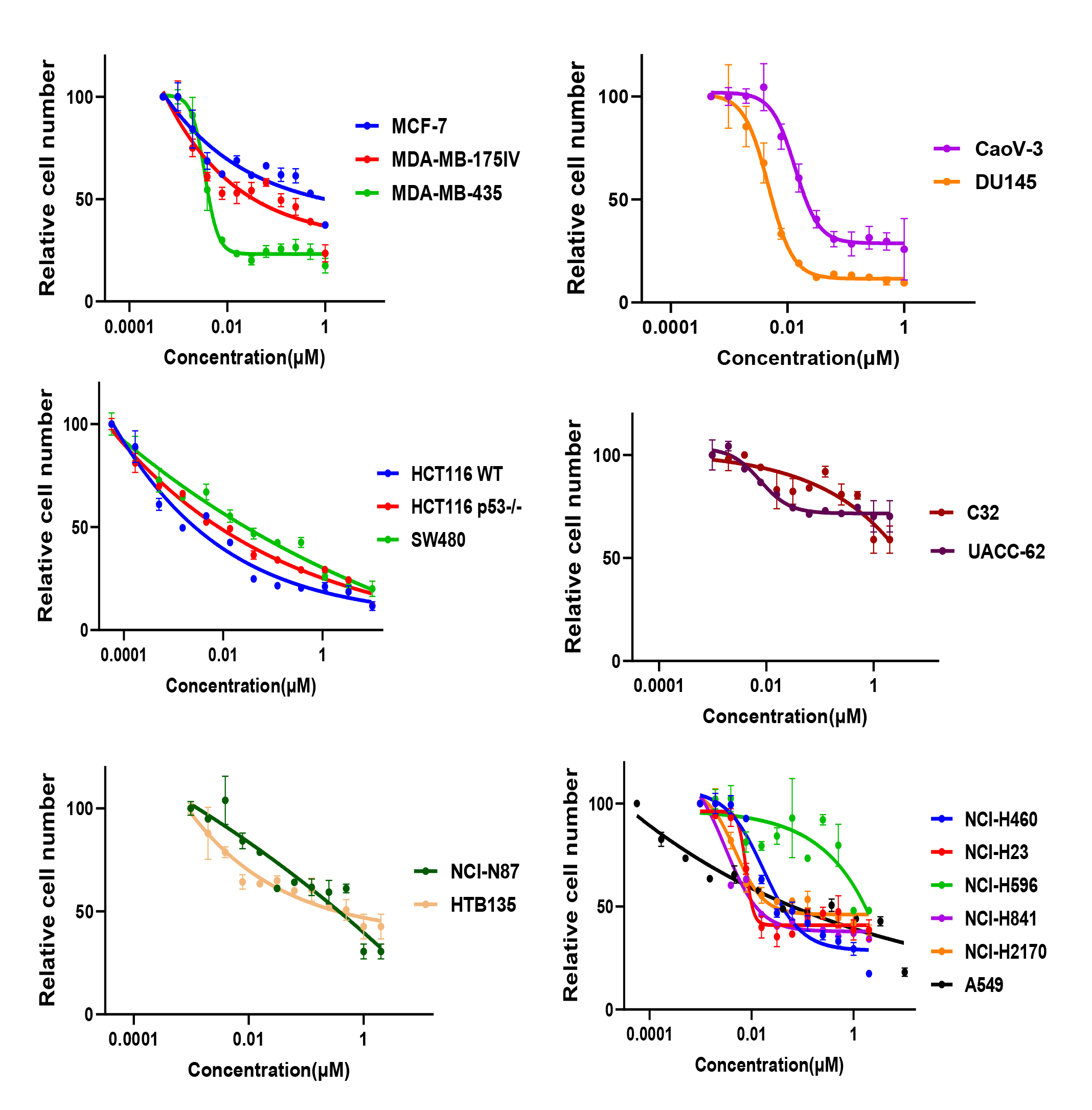

Supplement: S1 Fig — Each cell line was treated with LXY18 at a concentration range with 12 concentration points in a 2 or 3-fold dilution. The number of viable cells was determined by the MTT assay 72 h after the initiation of treatment. (TIF) [file pone.0293283.s001.tif]

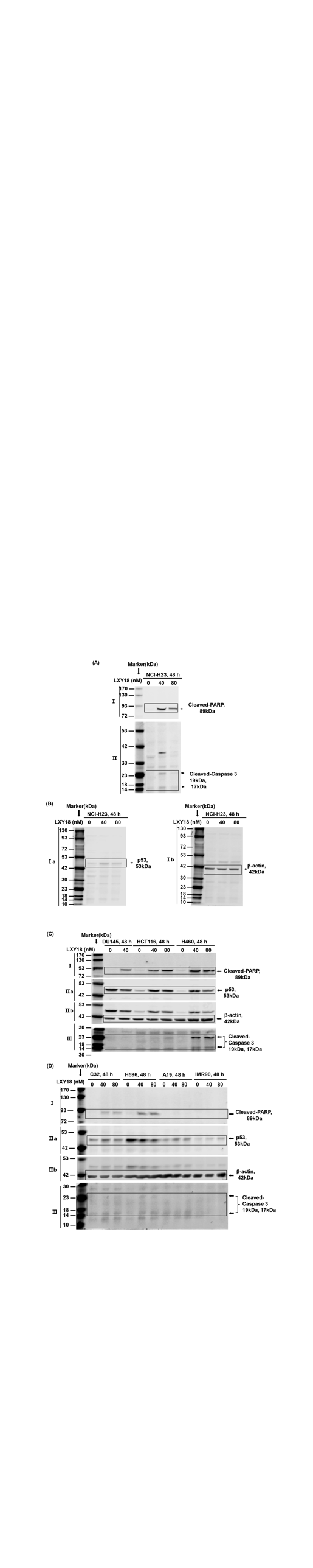

Supplement: S2 Fig — (A) One membrane was cut into the upper part (I) and the bottom part (II). The upper part was probed for cleaved-PARP (I), and the bottom part was probed for cleaved-caspase 3 (II). (B) One whole membrane was sequentially probed for p53 (Ia) and β-actin (Ib). (C, D) One membrane was cut into the upper part (I), the middle part (II), and the bottom part (III). The upper part was probed for cleaved-PARP (I), and the middle part (II) was sequentially probed for p53 (IIa) and β-actin (IIb). The bottom part was probed for cleaved caspase 3 (III). (TIF) [file pone.0293283.s002.tif]

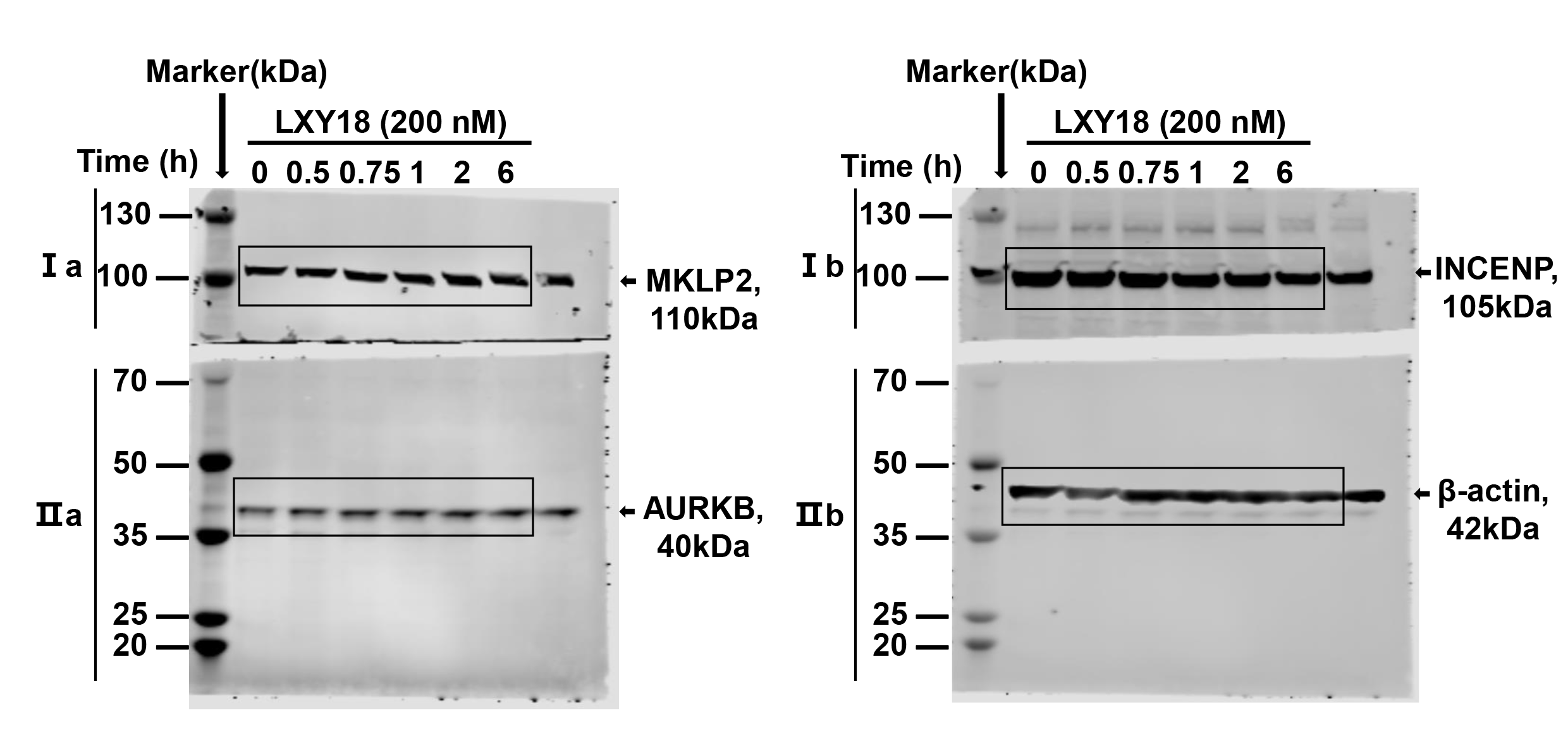

Supplement: S3 Fig — One membrane was cut into the upper part (I) and the bottom part (II). The upper part was sequentially probed for MKLP2 (Ia) and INCENP (Ib). The bottom part was sequentially probed for AURKB (IIa) and β-actin (IIb). (TIF) [file pone.0293283.s003.tif]

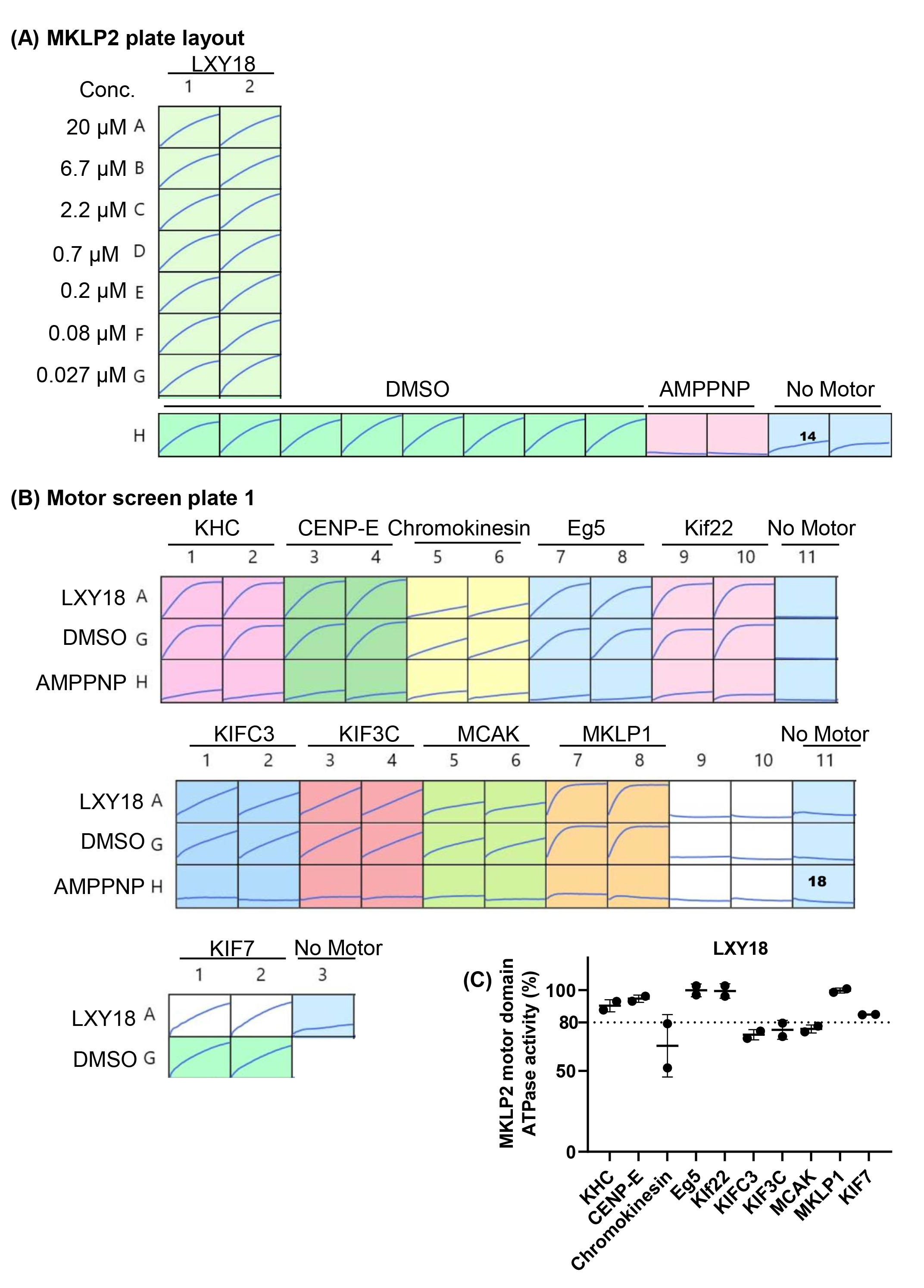

Supplement: S4 Fig — The microtubule-stimulated ATPase activity of the indicated motor proteins was determined in the presence of the indicated concentrations of LXY18. An ATP-competitive inhibitor AMPPNP at 1mM and a reaction mixture lacking motor proteins were used as positive controls for each test. DMSO, the solvent for LXY18, was used as a negative control. (TIF) [file pone.0293283.s004.tif]

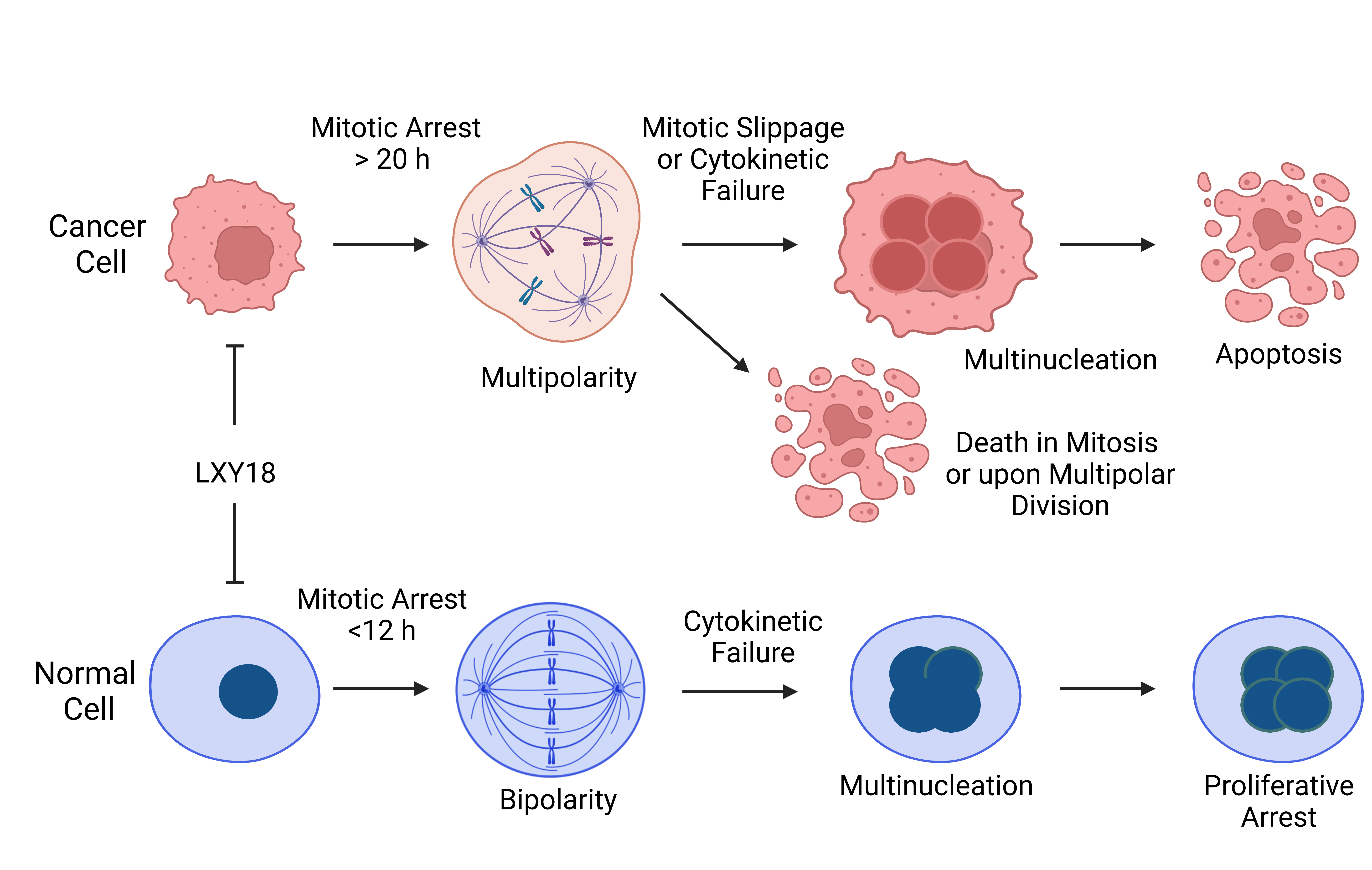

Supplement: S1 Graphical abstract — (TIF) [file pone.0293283.s009.tif]
