# Supplementary material for: The Aurora kinase B relocation blocker LXY18 triggers mitotic catastrophe selectively in malignant cells
Source: PLoS One. 2023 Oct 30;18(10):e0293283. doi: 10.1371/journal.pone.0293283 (PMC10615259; doi:10.1371/journal.pone.0293283)
Supplement: S1 Table — IC50 data are from the genomics of drug sensitivity in cancer website (https://www.cancerrxgene.org). (DOCX) [file pone.0293283.s005.docx]

**S1 Table. The IC_50_ of AURK inhibitors in different 11 human cancer cell lines in Depmap.** IC50 data are from the genomics of drug sensitivity in cancer website ([https://www.cancerrxgene.org](https://www.cancerrxgene.org/)).

| **IC50 (μM)** | **Cpd 10** | **Alisertib** | **ZM447439** |
| --- | --- | --- | --- |
| **A549** | 7.11 | 15.38 | 6.43 |
| **C32** | 385.54 | 19.25 | 12.44 |
| **DU145** | 164.58 | 4.21 | 35.53 |
| **HCT116** | 0.82 | 0.63 | 1.35 |
| **MCF7** | 130.34 | 0.53 | 17.05 |
| **NCI-H2170** | 23.08 | 34.42 | 5.89 |
| **NCI-H23** | 3.37 | 8.54 | 8.12 |
| **NCI-H460** | / | 23.29 | 3.31 |
| **NCI-H841** | 15.76 | 5.65 | 3.62 |
| **NCI-N87** | 79.18 | 11.66 | 54.74 |
| **UACC-62** | 125.93 | 31.21 | 8.67 |
