# Supplementary material for: The Aurora kinase B relocation blocker LXY18 triggers mitotic catastrophe selectively in malignant cells
Source: PLoS One. 2023 Oct 30;18(10):e0293283. doi: 10.1371/journal.pone.0293283 (PMC10615259; doi:10.1371/journal.pone.0293283)
Supplement: S2 Table — The table shows 18 human cancer cell lines examined, their related cancer types, and the precise oncogenic alterations recognized in each cell line. (DOCX) [file pone.0293283.s006.docx]

**S2 Table. Oncogenic driver mutations in 18 human cancer cell lines.** The table shows 18 human cancer cell lines examined, their related cancer types, and the precise oncogenic alterations recognized in each cell line.

| **No.** | **Type** | **Cell line** | **Oncogenic Alterations** |  |
| --- | --- | --- | --- | --- |
| 1 | Breast | MCF7 | PIK3CA, c.1633G>A, p.E545K; |  |
| 2 | Breast | MDA-MB-175IV | [PIK3C2B](https://depmap.org/portal/gene/PIK3C2B), p.C561F. |  |
| 3 | Breast | MDA-MB-435S | AKT2, c.1138C>T, p.L380L; PIK3CB, c.1604T>G, p.L535R. ATR, c.1802C>T, p.S601F |  |
| 4 | Ovary | CaoV-3 | TP53,c.406C>T, p.Q136*. ATM, c.5706_5707insA, p.K1903fs MYCN, c.1152A>C, p.R384S |  |
| 5 | Prostate | DU145 | AKT2, c.395C>T, p.T132M; PIK3CB, c.2056G>A, p.A686T, c.30T>A, p.A10A. TP53,c.820G>T, p.V274F; c.668C>T, p.P223L. BRAF, c.2124C>T, p.P708P. RB1, c.2143A>T, p.K715*;. MYCL, c.835G>T, p.E279*; |  |
| 6 | Colon | HCT116 | PIK3CA, c.3140A>G, p.H1047R. BRAF,c.1998T>C, p.I666I; c.903A>G, p.P301P; ; ARAF, c.758delC, p.T253fs. KRAS, c.38G>A, p.G13D. ATM, c.3380C>T, p.A1127V. Met, c.710delT, p.V237fs |  |
| 7 | Colon | HCT116 p53-/- | TP53 deletion. PIK3CA, c.3140A>G, p.H1047R. BRAF,c.1998T>C, p.I666I; c.903A>G, p.P301P; ; ARAF, c.758delC, p.T253fs. KRAS, c.38G>A, p.G13D. ATM, c.3380C>T, p.A1127V. Met, c.710delT, p.V237fs. |  |
| 8 | Colon | SW480 | TP53,c.925C>T, p.P309S; c.818G>A, p.R273H. KRAS,c.35G>T, p.G12V. ATM, c.7382G>C, p.R2461P |  |
| 9 | Skin | UACC-62 | PTEN, c.740_741insA, p.P248fs. BRAF, c.1799T>A, p.V600E |  |
| 10 | Skin | C32 | PIK3CB, c.2920G>A, p.G974R. BRAF, c.1799T>A, p.V600E. CDKN2A deletion  PTEN deletion |  |
|  |  |  |  |  |
| 11 | Stomach | NCI-N87 | TP53, R248Q. |  |
| 12 | Stomach | HTB135 | TP53,c.955A>T, p.K319*. |  |
| 13 | Lung | NCI-H460 | PIK3CA, c.1633G>A, p.E545K. KRAS,c.183A>T, p.Q61H. |  |
| 14 | Lung | NCI-H23 | TP53,c.738G>C, p.M246I. KRAS, c.34G>T, p.G12C. ATM, c.5756A>C, p.Q1919P. |  |
|  |  |  |  |  |
| 15 | Lung | NCI-H596 | PIK3CA, c.1633G>A, p.E545K. TP53,c.733G>T, p.G245C. RB1,c.541_542insT, p.I181fs |  |
|  |  |  |  |  |
| 16 | Lung | NCI-H841 | TP53, c.725G>C, p.C242S. |  |
| 17 | Lung | NCI-H2170 | PIK3CA, c.472C>G, p.R158G. ATR, c.6568C>T, p.R2190C |  |
| 18 | Lung | A549 | KRAS, c.34G>A, p.G12S |  |
