# Supplementary material for: The Aurora kinase B relocation blocker LXY18 triggers mitotic catastrophe selectively in malignant cells
Source: PLoS One. 2023 Oct 30;18(10):e0293283. doi: 10.1371/journal.pone.0293283 (PMC10615259; doi:10.1371/journal.pone.0293283)
Supplement: S4 Table — The table provides comprehensive information regarding several types of items used in the experiment, including reagents, antibodies, software, and equipment. (DOCX) [file pone.0293283.s008.docx]

**S4 Table.** **Reagents, antibodies, software and equipment’s information.** . The table provides comprehensive information regarding several types of items used in the experiment, including reagents, antibodies, software, and equipment.

| Reagents | Source | Catalogue Number |
| --- | --- | --- |
| Antibodies | | |
| Rabbit anti-cleaved Caspase-3 (Asp175) | CST | 9661L |
| Rabbit anti-Cleaved PARP (Asp214) | CST | 9541 |
| Rabbit anti-p53 | CST | 9282 |
| Mouse anti-β-actin | Proteintech | 66009-1-1g |
| Rabbit anti-AURKB | Absin | 131460 |
| Rabbit anti-inner centromere protein (INCENP) polyclonal antibody | A gift From T. Stukenberg (University of Virginia, Charlottesville) and W. C. Earnshaw (University of Edinburgh, Edinburgh) | |
| Human autoimmune serum CREST | Reference[1] | |
| Mouse anti-MKLP2 | Santa Cruz | SC-374508 |
| IRDye® 800CW Goat anti-Mouse IgG | Li-cor | 926-32210 |
| IRDye® 680RD Goat anti-Rabbit IgG | Li-cor | 926-68071 |
| Chemicals and Reagents | | |
| AMG900 | TargetMol | 945595-80-2 |
| AZD1152 | Selleck | S1147 |
| MLN8237 | Selleck | S1133 |
| LXY18 | Made in-house | Reference[2] |
| 11i | Made in-house | Reference[1] |
| Z-VAD-fmk | Targetmol | 187389-52-2 |
| aphidicolin | Aladdin | 38966-21-1 |
| DMEM | Gibco | 12100061 |
| Fetal bovine serum (FBS) | Excell | FSP500 10099141 |
| Penicillin-Streptomycin | Thermo Fisher | 15140122 |
| L-glutamine | Thermo Fisher | 25030081 |
| Sodium pyruvate | Thermo Fisher | 11360070 |
| DMSO | Aladdin | D103277 |
| Commercial Kits | | |
| Myco-Lumi™ Luminescent Mycoplasma Detection Kit | Beyotime | C0298M |
| Experimental Models | | |
| Software and Algorithms | | |
| GraphPad Prism 8.0.2 | GraphPad software | https://www. GraphPad.com/ |
| Equipment | | |
| Evos FL Auto | Thermo | NA |
| Odyssey CLx | Li-cor | NA |

**References**

1. Lv G, Shi Q, Zhang T, Li J, Kalashova J, Long Y, et al. 2-Phenoxy-3, 4′-bipyridine derivatives inhibit AURKB-dependent mitotic processes by disrupting its localization. Eur J Med Chem. 2022; 114904. doi:10.1016/J.EJMECH.2022.114904

2. Li J, Zhang T, Shi Q, Lv G, Zhou X, Choudhry N, et al. An orally bioavailable 4-phenoxy-quinoline compound as a potent AURKB relocation blocker for cancer treatment. bioRxiv. 2023; 2023.01.29.526078. doi:10.1101/2023.01.29.526078
