# Supplementary material for: The Aurora kinase B relocation blocker LXY18 triggers mitotic catastrophe selectively in malignant cells
Source: PLoS One. 2023 Oct 30;18(10):e0293283. doi: 10.1371/journal.pone.0293283 (PMC10615259; doi:10.1371/journal.pone.0293283)

MAIN FIGURE
ORIGINAL BLOT

Main Fig 3 original blot in Fig S2_A-D

Fig S2_A-D

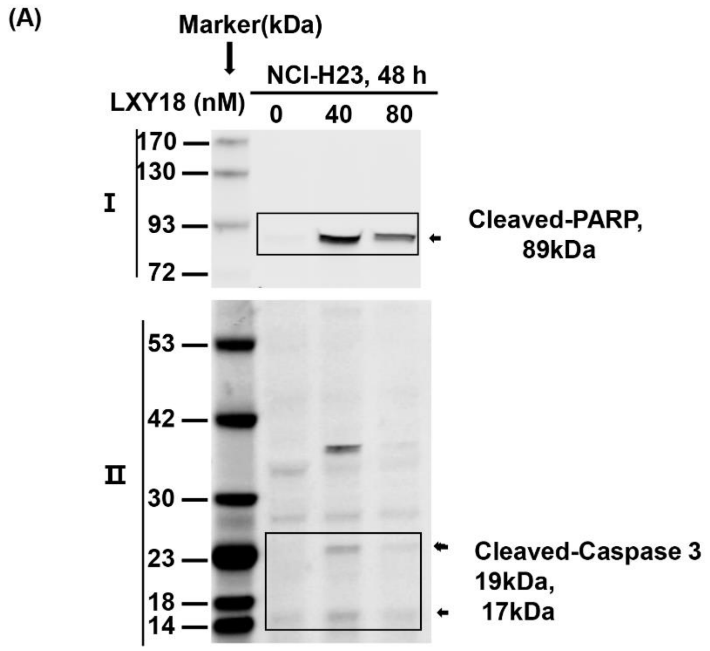

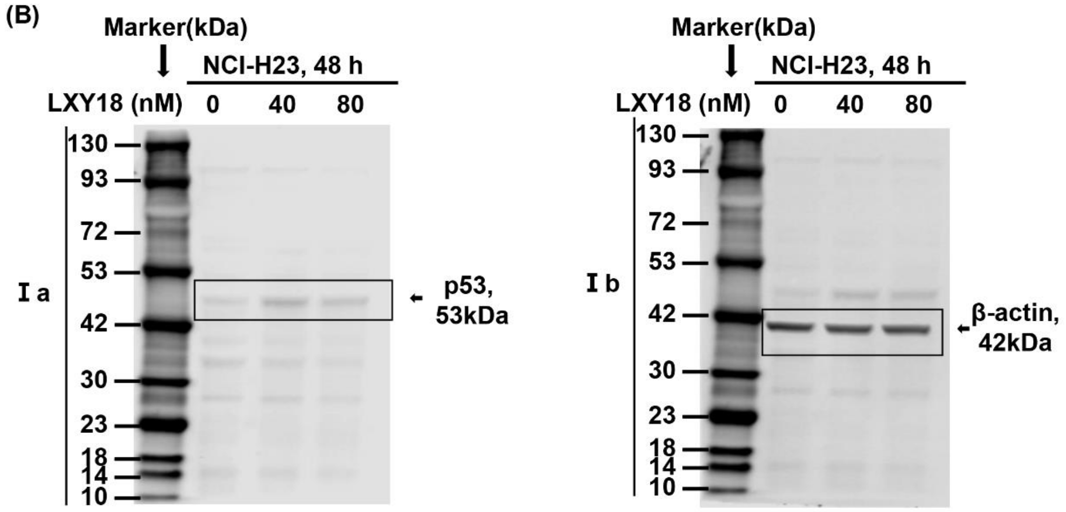

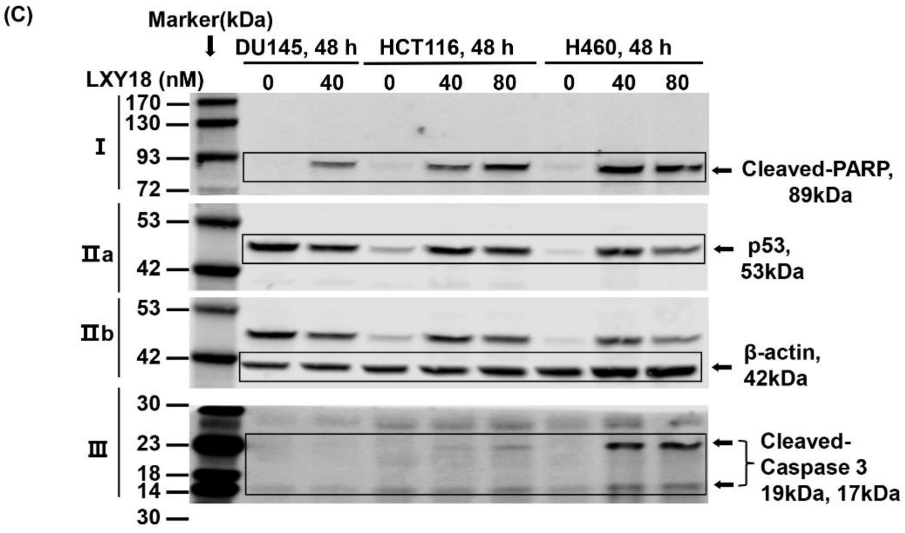
(C)
Marker(kDa)
DU145, 48 h
HCT116, 48 h
H460, 48 h
LXY18 (nM)
0 40 0 40 80 0 40 80
170
130
93
72
53
42
53
42
30
23
18
14
30
I
IIa
IIb
III
Cleaved-PARP, 89kDa
p53, 53kDa
β-actin, 42kDa
Cleaved-Caspase 3 19kDa, 17kDa

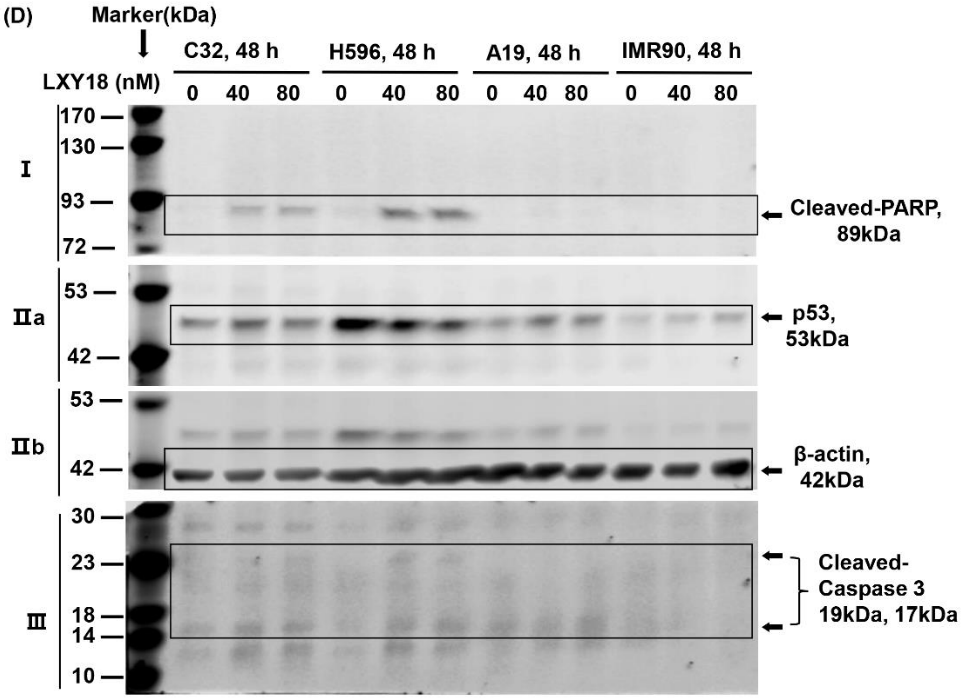
(D)
Marker(kDa)
C32, 48 h
H596, 48 h
A19, 48 h
IMR90, 48 h
LXY18 (nM)
0 40 80 0 40 80 0 40 80 0 40 80
170
130
93
72
53
42
53
42
30
23
18
14
10
I
IIa
IIb
III
Cleaved-PARP, 89kDa
p53, 53kDa
β-actin, 42kDa
Cleaved-Caspase 3 19kDa, 17kDa

Main Fig 7 original blot in Fig S3

Fig S3

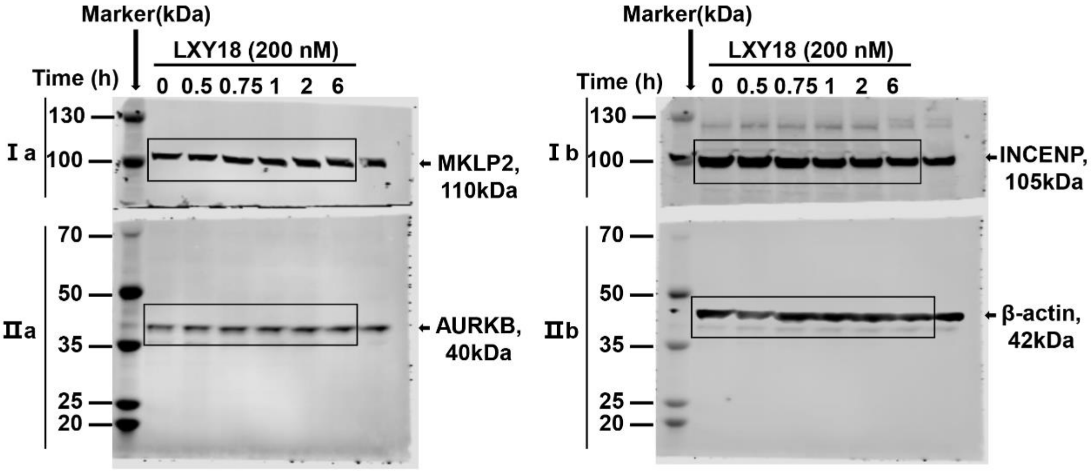

Supplementary Figs S2 and S3 original blots

S2_Fig A
Cleaved-PARP, 89kDa

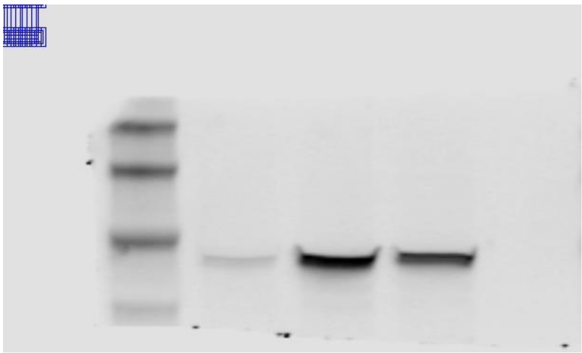

Cleaved-caspase3, 17kDa,19kDa

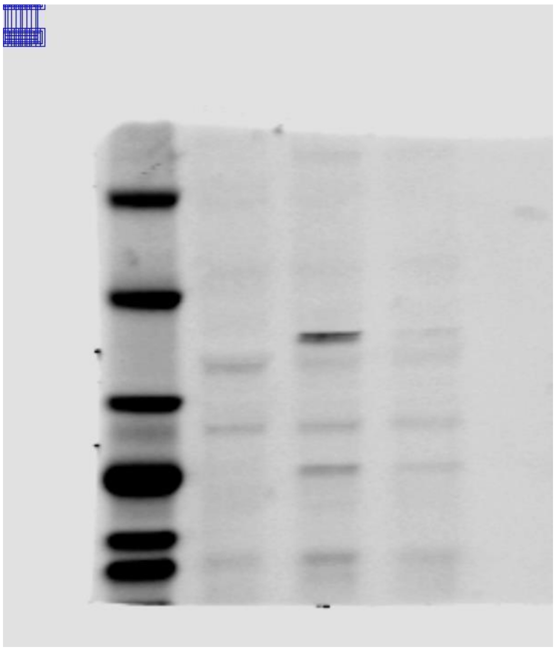

S2_Fig B

p53, 53kDa

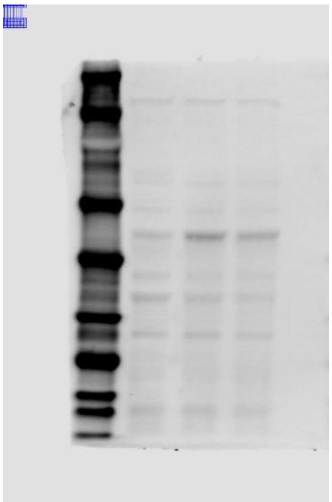

β-actin, 42kDa

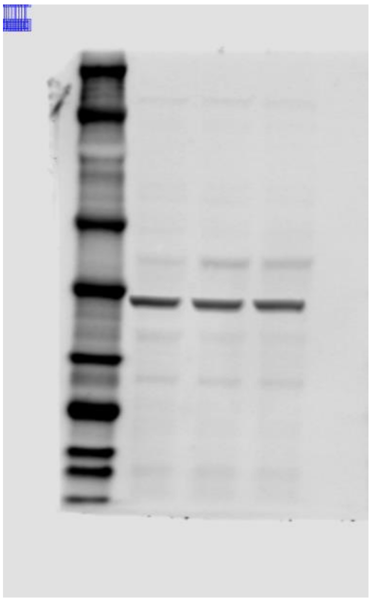

S2_Fig C

Cleaved-PARP, 89kDa

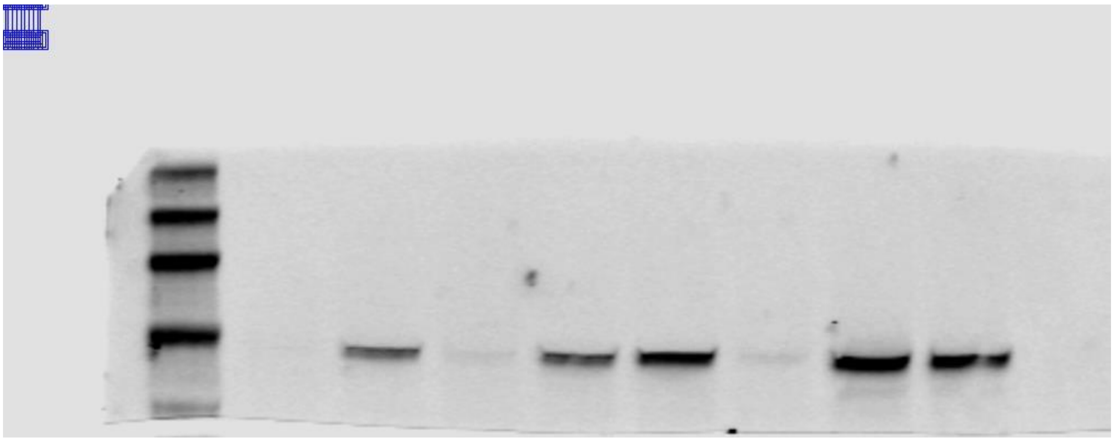

p53, 53kDa

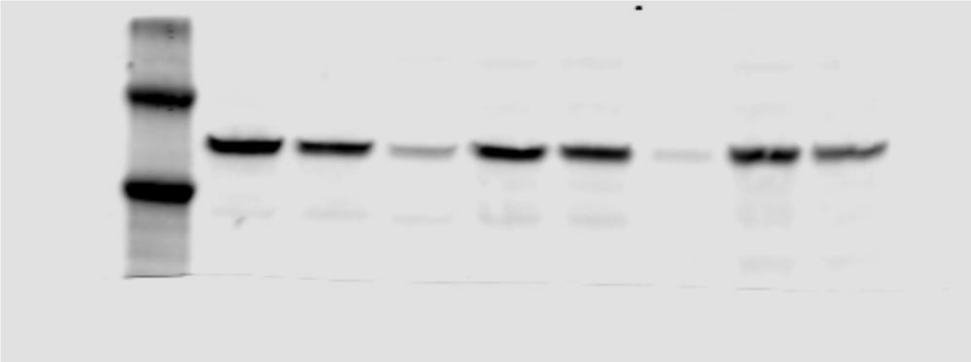

β-actin, 42kDa

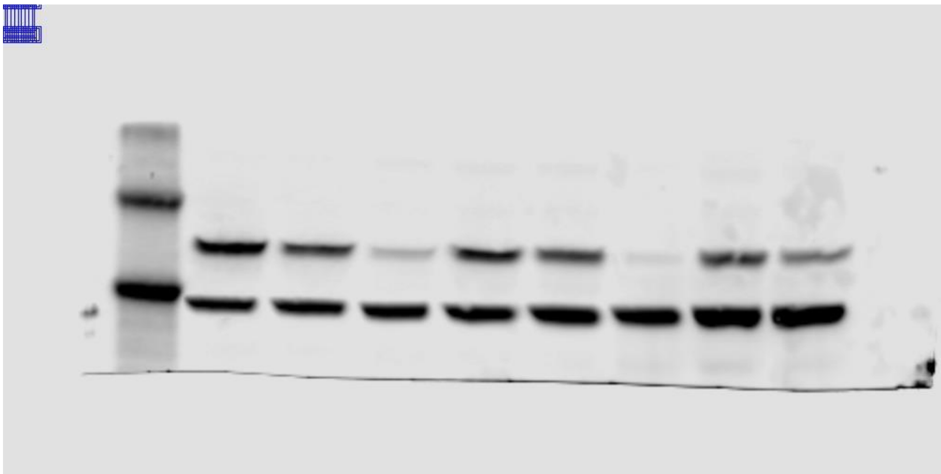

Cleaved-caspase3, 17kDa,19kDa

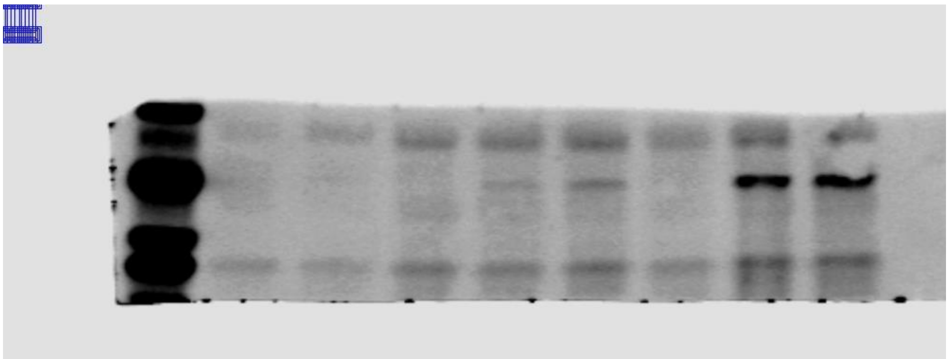

S2_Fig D

Cleaved-PARP, 89kDa

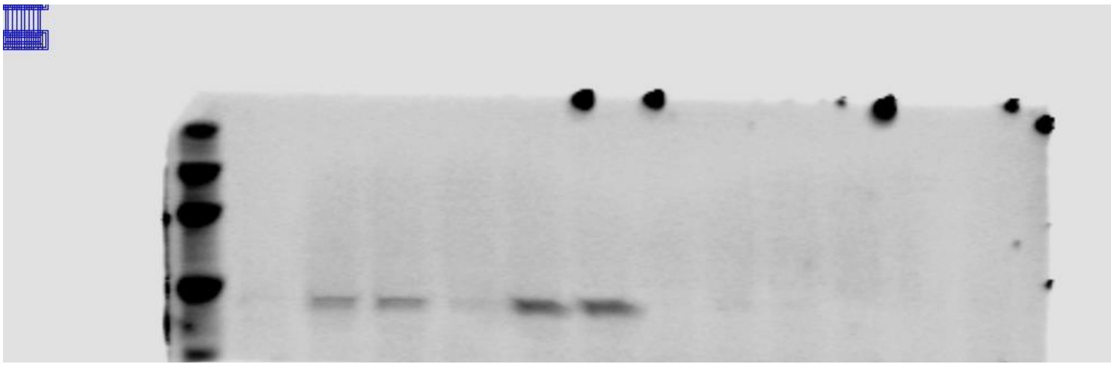

p53, 53kDa

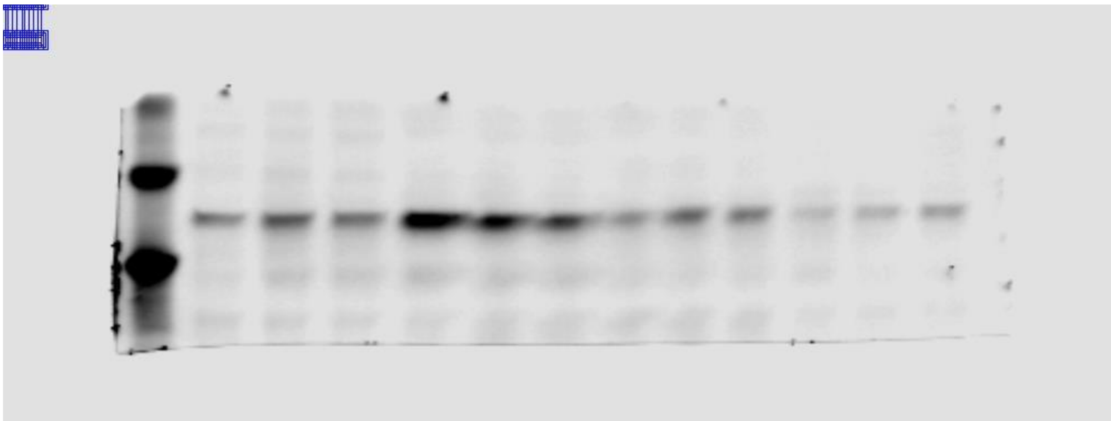

β-actin, 42kDa

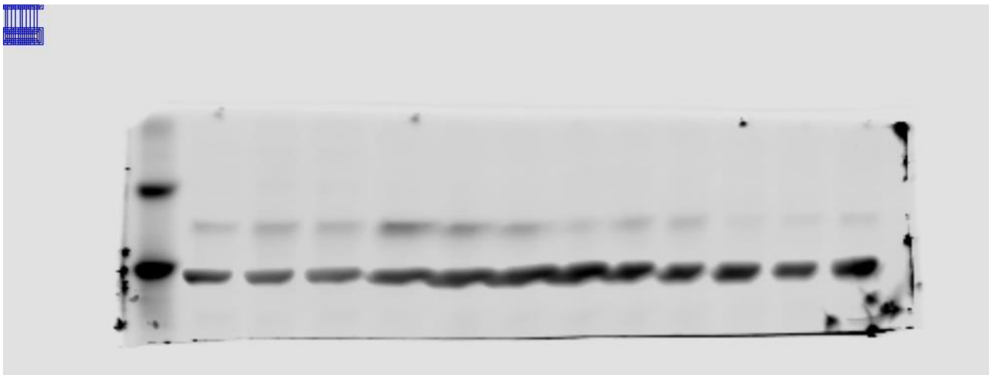

Cleaved-caspase3, 17kDa,19kDa

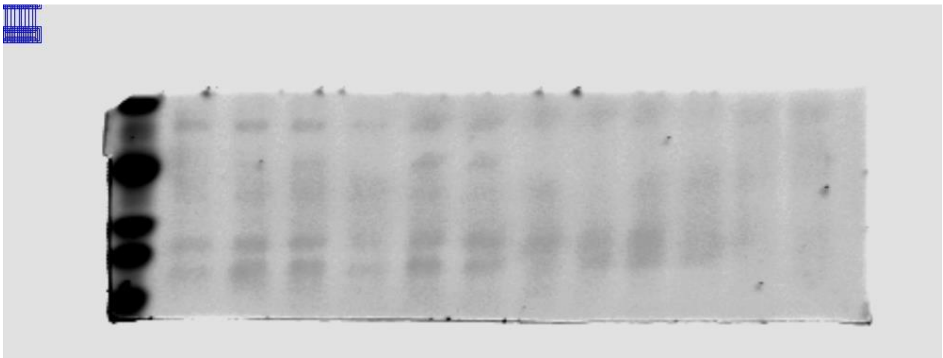

S3_Fig

MKLP2, 110kDa

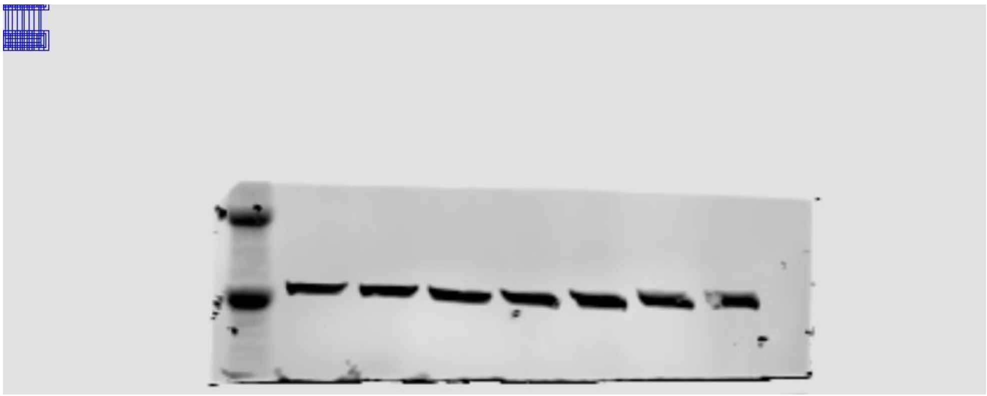

AURKB, 40kDa

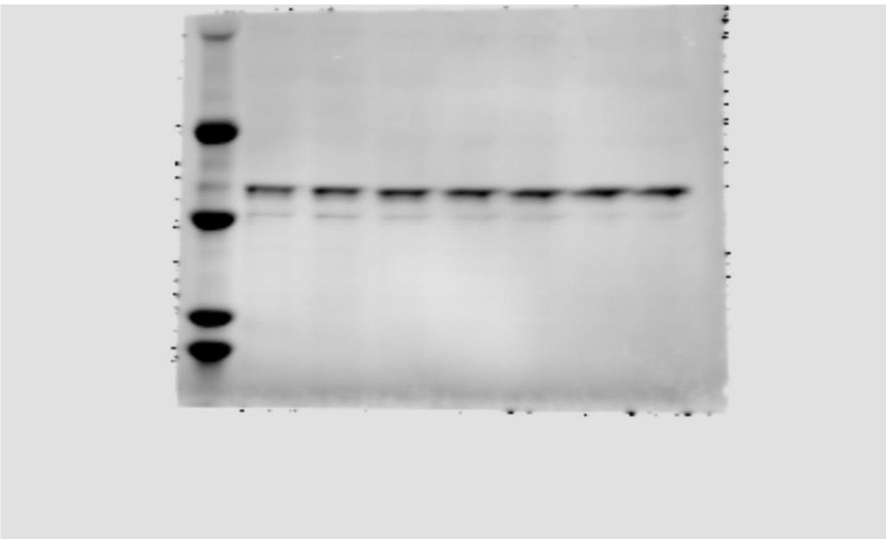

INCENP, 105kDa

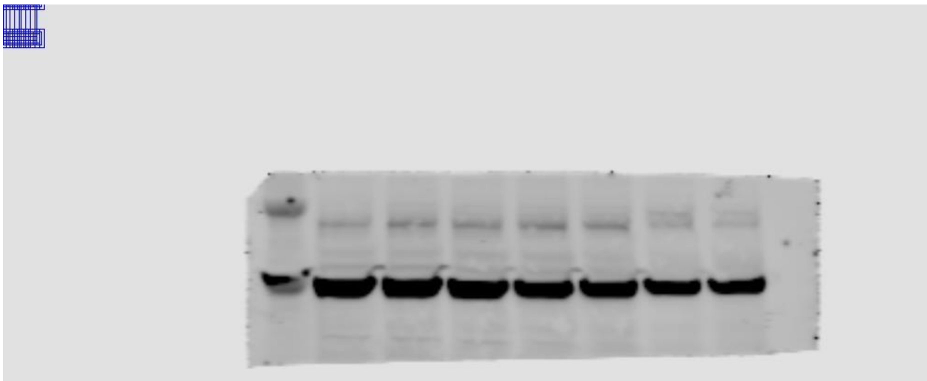

β-actin, 42kDa

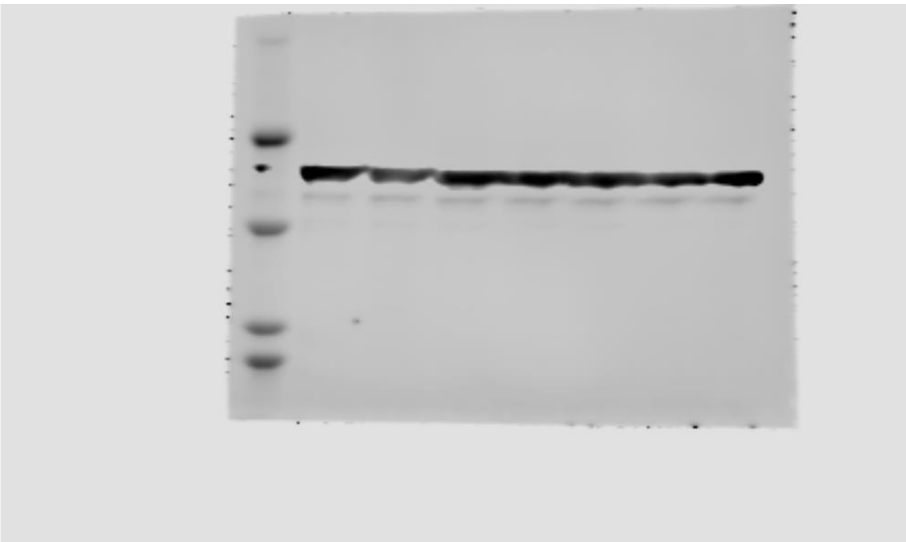

Supplement: S1 Raw images — (PDF) [file pone.0293283.s010.pdf]
